# Exploring the Use of Cold Atmospheric Plasma to Overcome Drug Resistance in Cancer

**DOI:** 10.3390/biomedicines11010208

**Published:** 2023-01-14

**Authors:** Dzohara Murillo, Carmen Huergo, Borja Gallego, René Rodríguez, Juan Tornín

**Affiliations:** 1 Instituto de Investigación Sanitaria del Principado de Asturias (ISPA), Hospital Universitario Central de Asturias, 33011 Oviedo, Spain; 2 Instituto Universitario de Oncología del Principado de Asturias, 33006 Oviedo, Spain; 3 CIBER en Oncología (CIBERONC), 28029 Madrid, Spain

**Keywords:** cold atmospheric plasma, drug resistance, plasma oncology

## Abstract

Drug resistance is a major problem in cancer treatment, as it limits the effectiveness of pharmacological agents and can lead to disease progression. Cold atmospheric plasma (CAP) is a technology that uses ionized gas (plasma) to generate reactive oxygen and nitrogen species (RONS) that can kill cancer cells. CAP is a novel approach for overcoming drug resistance in cancer. In recent years, there has been a growing interest in using CAP to enhance the effectiveness of chemotherapy drugs. In this review, we discuss the mechanisms behind this phenomenon and explore its potential applications in cancer treatment. Going through the existing literature on CAP and drug resistance in cancer, we highlight the challenges and opportunities for further research in this field. Our review suggests that CAP could be a promising option for overcoming drug resistance in cancer and warrants further investigation.

## 1. Introduction

Drug resistance is a major problem in cancer treatment, as it limits the effectiveness of pharmacological agents and can lead to disease progression. Current cancer treatments often involve the use of chemotherapeutics and other xenobiotic compounds that target specific molecular pathways involved in cancer cell growth and survival [1]. Unfortunately, some cancer cells become resistant to these drugs, allowing them to grow and divide despite treatment. This can lead to the development of more aggressive and difficult-to-treat tumors, which is one of the major challenges faced in the fight against cancer [2].

One potential approach to overcoming drug resistance in cancer is the use of novel therapeutic strategies such as cold atmospheric plasma (CAP). CAP is a developing technology that uses ionized gas (plasma) to generate reactive oxygen and nitrogen species (RONS) to eliminate cancer cells. In preclinical studies, CAP has been shown to increase the efficacy of various anticancer drugs, including chemotherapeutics and target agents, in cancer cell lines and animal models. This is thought to occur through a combination of mechanisms, including the direct killing of cancer cells, reversal of drug resistance, and induction of anti-tumor immunity.

In addition to its potential use in cancer treatment, CAP has been studied for various biomedical applications, including wound healing, sterilization, and tissue engineering [3]. CAP generates RONS that can kill bacteria and other microorganisms, making it a potentially effective tool for sterilization and infection control. Additionally, CAP has been shown to promote the growth and differentiation of various cell types, including skin and nerve cells, which could have applications in tissue engineering and regenerative medicine.

Overall, the use of CAP as a potential combinatorial treatment to mitigate tumor burden and overcome drug resistance in cancer is a promising area of research, with the potential to improve the effectiveness of existing anticancer drugs and develop new therapeutic strategies. However, further research is needed to completely understand the mechanisms by which CAP eliminates drug resistance and to develop safe and effective clinical treatments using this technology.

### 1.1. An Overview of Drug Resistance in Cancer

Cancer is a leading cause of death worldwide, and one of the major challenges in its treatment is drug resistance. This refers to the ability of cancer cells to withstand the effects of chemotherapy drugs, resulting in treatment failure and disease progression. Drug resistance can arise through various mechanisms, including changes in the expression of drug target proteins, the activation of cell survival pathways, and the acquisition of mutations that confer resistance [2]. Efforts to overcome drug resistance in cancer have focused on developing new pharmacological agents and combination therapies, as well as deciphering and targeting the mechanisms of resistance. Despite these efforts, drug resistance remains a major obstacle in cancer treatment, and improving our understanding of this phenomenon will allow us to develop effective strategies to overcome it. 

Drug resistance can occur through two main mechanisms: intrinsic and extrinsic. Intrinsic drug resistance refers to the inherent resistance of cancer cells to a particular drug, which may be due to differences in the genetic makeup of tumor cells or the presence of specific molecular pathways that protect cells from certain effects of the drugs. Intrinsic resistance is typically observed when a drug is first tested in a subset of cancer cells and can occur even in the absence of prior exposure to the drug.

Extrinsic drug resistance, on the other hand, is acquired resistance that develops over time as a result of drug exposure. This type of resistance is often due to changes in cancer cells that allow them to survive and proliferate despite the presence of the drug. These changes may include the activation of signaling pathways that protect cells from the drug’s effects, the upregulation of drug efflux pumps that eliminate the drug from the cells, or the development of mutations in the drug target that render it less sensitive to the compound.

Cancer cells can develop drug resistance through a variety of mechanisms. Some of those include [4]:

- Changes in the drug target: cancer cells can acquire mutations at the target site of the drug that make them less sensitive to the drug.
- Activation of protective signaling pathways: upregulating pathways that promote cell survival or inhibiting those that promote cell death.
- Expression of drug efflux pumps in cancer cells: proteins that actively transport drugs out of the cell and prevent them from reaching their targets.
- The presence of cells with stem cell-like properties: cancer stem cells (CSC) are a subset of cells that are less sensitive to the current drugs, most likely because they usually present lower levels of drug targets or better mechanisms of DNA damage repair.
- The tumor microenvironment: which includes the surrounding stroma and immune system and can provide a protective environment for cancer cells making them less sensitive to xenobiotic compounds. The stroma may produce growth factors that support the survival of cancer cells.

### 1.2. Drug Resistance: Challenges and Limitations of Current Strategies

Intrinsic and acquired resistance mechanisms coexist during tumor evolution and can vary greatly, as detailed in [2,4]. Importantly, the mechanisms of acquired drug resistance can be utterly different from the pre-existing intrinsic drug resistance or involve the selective expansion of intrinsic drug resistance. Therefore, developing novel therapeutic options to overcome drug-resistant phenotypes in cancer is a pressing need; however, less than 5% of new anti-tumor drugs succeed in clinical trials [5,6,7,8]. 

Several approaches to overcome drug resistance in cancer are being explored:

- Combination therapy: the use of multiple drugs to simultaneously attack different pathways or targets in cancer cells, which may help prevent the development of resistance to a single drug.
- Targeting drug resistance mechanisms: using specific drugs or agents targeted to resistance mechanisms such as drug efflux pumps or signaling pathways.
- Repurposing existing drugs: identifying new uses for existing drugs that were originally developed for other indications, such as repurposing antibiotics to treat cancer.
- Novel therapeutic strategies: developing new therapeutic approaches, such as immunotherapy or gene therapy, to attack tumor cells in ways that may be less susceptible to resistance.
- Personalized medicine: deciphering genetic or molecular profiling of cancer cells to identify individualized treatment strategies that are tailored to the specific characteristics of a patient’s cancer and are less likely to be affected by resistance.
- Novel drug delivery systems: exploring advanced drug delivery systems, such as nanoparticles or targeted carriers, to improve the delivery of anti-tumor compounds and reduce the potential for resistance.
- Novel drug combinations: testing new combinations of existing drugs or novel agents that may show synergistic effects, thus solving drug resistance to a single agent.

However, current approaches to combating drug resistance in cancer have shown several limitations and challenges [4]. For instance, one major limitation of combination therapy is the difficulty in identifying optimal combinations of drugs that are effective against a particular type of cancer. In addition, combining drugs may result in greater side effects than those observed for a single drug, affecting patients’ well-being. Regarding the use of novel drugs specifically targeted to the mechanisms of drug resistance, one disadvantage is that they may not be effective against all types of drug resistance. In addition, targeted agents can be expensive and may not be widely available, limiting their use.

The challenges posed by these existing strategies for overcoming drug resistance in cancer have long been a major concern in the medical community, which has not ceased the search for new therapeutic approaches. Recently, cold atmospheric plasma (CAP), has gained attention as a promising option for tackling this issue.

## 2. Plasma, the Fourth State of Matter

The vast majority of the observed mass in the universe is composed of plasma, defined as a quasi-neutral ionized gas produced by an electrical discharge and composed of photons, electrons, ions, reactive oxygen and nitrogen species (RONS), UV, visible light, and electromagnetic radiation [9]. Plasma can occur naturally, as in lightning and the northern lights, or artificially, by applying a strong electrical field to a gas. The use of plasma in industrial and biomedical applications is continuously and rapidly growing. In plasma devices that are being developed, the electrical discharge breaks the bond between the nuclei and electrons of atoms, ionizing the gas and resulting in a high-energy state of matter. The temperature of plasma depends on the degree of ionization and is crucial in determining its use.

**Figure 1:**
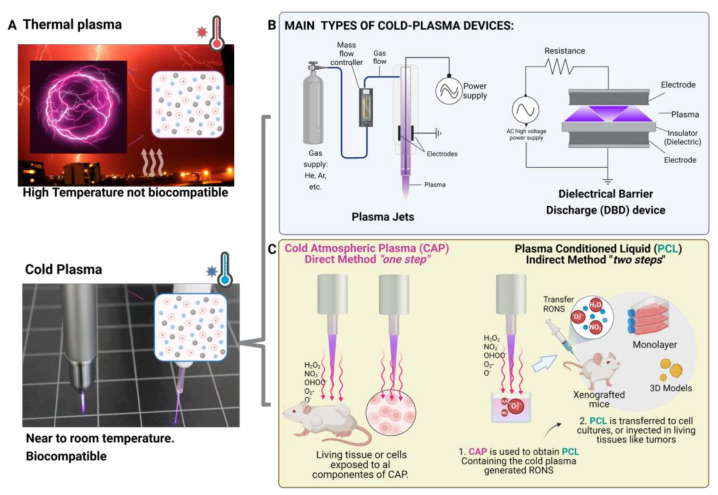
Overview of cold atmospheric plasma. (**A**) Atmospheric plasma can be divided into two categories: thermal plasma, which operates at nonbiocompatible temperatures (e.g., lighting), and cold atmospheric plasma (CAP), which operates near room temperature and is therefore suitable for biomedical applications. (**B**) There are two main types of CAP devices used in cancer research: plasma jets and dielectric barrier discharge. (**C**) These devices can be applied to tissues or cell cultures in two ways: directly, named as CAP; where all the components of the plasma act against the tissue or cells (left), or indirectly, where a plasma-conditioned liquid (PCL) containing the main cytotoxic components of the CAP (plasma-generated reactive oxygen and nitrogen species, or RONS) is obtained and applied to the tissue or cells (right). Figure created using Biorender.com.

Hot thermal plasma refers to a type of plasma that is produced at a high temperature, typically in the range of several thousand degrees Celsius (Figure 1A). This can be achieved by heating a gas to a high temperature using an electric arc, a laser, or some other form of energy input. Hot thermal plasmas are often used in welding, cutting, and surface treatment applications, as well as in the production of materials, such as ceramics and refractory metals. 

Non-thermal plasma refers to a type of plasma that is produced at a temperature that is significantly lower than the thermal equilibrium temperature of the gas. This can be achieved through various methods, such as applying high-frequency electric fields, using microwaves, or using lasers. Non-thermal plasmas are often produced at temperatures in the range of several hundred to several thousand degrees Celsius, depending on the specific method used to generate the plasma. Non-thermal plasmas have a wide range of applications, including surface cleaning and modification, air and water purification, and medicine. They are also used in the production of semiconductors, the synthesis of nanomaterials, and the generation of X-rays.

### 2.1. Cold-Plasma for Medicine

#### 2.1.1. Cold-Plasma Devices

CAP is a type of non-thermal plasma that is generated at relatively low temperatures (near room temperature). CAP can be generated using various devices, such as dielectric barrier discharge (DBD) devices or plasma jets [3] (Figure 1B). Two main types of CAP devices have been described in the literature on plasma medical research: dielectric barrier discharge devices (DBDs) and plasma jets (Figure 1B). DBDs consist of two parallel electrodes with a dielectric material placed between them to prevent direct electrical contact. This device generates a direct discharge on a biological sample [10], which is placed between a high-voltage electrode and a grounded electrode. High voltages are necessary to produce the discharge required to create plasma. The resulting CAP has diameters of up to 200 μm and can propagate randomly over the entire electrode surface, depending on the excitation voltage and frequency [11,12]. 

Plasma jets, on the other hand, use a high-voltage electrical discharge to generate a stream of plasma that can be directed towards a specific target. These devices are also known as plasma needles or torches and are designed using a cavity-based arrangement containing a guided stream of working gas, frequently consisting of helium, air, or argon [13,14]. The plasma is ignited within the cavity between the two electrodes, and the resulting beam, known as the “effluent,” is directed towards the sample of interest. The effluent typically has diameters of 1–5 mm and lengths of up to a few centimeters. One important aspect of plasma jet devices is that the biological sample does not act as an electrode. This means that the CAP is transported by the gas flow from the discharge zone to the living cells or tissue, rather than being generated directly on or within the sample itself. This allows for more precise control over the plasma exposure and can help minimize the potential for damage to the sample.

DBD devices and plasma jets are both types of plasma generators that can be used to produce CAP to be applied to biological samples. However, there are some key differences between them [3,15,16]:

- Sample location: In a DBD device, the sample acts as an electrode and is typically located within the plasma discharge region, whereas in a plasma jet, the sample is typically located outside the plasma discharge region, downstream from the nozzle or orifices.
- Plasma production: DBD devices use high-voltage electric fields to ionize gas and produce plasma within an enclosed chamber. Plasma jets use a similar principle, but the plasma is generated within a nozzle or a series of orifices and is expelled as a jet or beam.
- Plasma characteristics: The plasma produced by a DBD device is typically more homogeneous and uniform than the plasma produced by a plasma jet, which tends to be more directional and focused.
- Limitations: DBD devices are generally limited to the production of plasma at low pressures (atmospheric or below), whereas plasma jets can be used to produce plasma at higher pressures. DBD devices are also limited in the types of gases that can be used, as some gases may not be stable under the high-voltage conditions required for plasma production. Plasma jets may be less efficient at producing plasma than DBD devices, as a significant portion of the plasma is lost through the nozzle or orifices.
- Advantages: Plasma jets offer several advantages for in vitro and in vivo research, including their small size, ease of handling, and ability to treat small volumes of liquid. Based on these properties and features, plasma jet devices may be more suitable for gentle treatment of a small area of a sample, whereas DBD devices are relatively simple and compact and can be used to produce a wide range of plasma species. Plasma jets are highly directional and can be used to produce a focused plasma beam that can be used for other many applications.

#### 2.1.2. Plasma Medicine

CAP has a wide range of biomedical applications due to its ability to produce a variety of reactive species, such as radicals and ions, that can interact with biological materials. CAP has been shown to have antimicrobial, antiviral, and anti-inflammatory effects and has been used to treat a variety of conditions, including wounds, infections, and cancer. CAP has also been used to inactivate pathogens in food and water and to decontaminate surfaces. CAP has been effective in eliminating a variety of dangerous pathogenic microorganisms [17,18], including antibiotic-resistant bacteria, fungi, and viruses [19], such as SARS-CoV-2 [20]. In fact, among the wide variety of CAP functions, it has also proven to be useful in dentistry for the treatment of oral pathogens, such as *Candida albicans* [21] and *Staphylococcus aureus* [17].

In addition to its antimicrobial properties, the role of CAP in promoting wound healing has been widely described [22,23,24]. CAP contributes to the removal of infections while simultaneously increasing cutaneous microcirculation [25], controlling blood coagulation [26], activating the immune system, and promoting the migration of keratinocytes and fibroblasts to the treated area [27].

Moreover, CAP has been shown to improve the adhesion and proliferation of osteoblasts, which can enhance the osteointegration of dental implants [28,29,30]. Researchers have also investigated the potential use of CAP in regenerative medicine. CAP treatment has been shown to promote regeneration of the nasal mucosa in vitro and in vivo [31], as well as chondrogenesis and endochondral ossification [32]. In addition, positive effects of CAP have been observed in the regeneration of several neural cell types, most likely due to its ability to enhance the stem cell properties of bone marrow, adipose, and neural stem cells [33,34].

### 2.2. Cold Plasma-Induced Oxidative Stress as a Cancer Therapy

In recent years, CAP has been demonstrated to be effective in eliminating human cancer cells in vitro and in vivo [11], as well as in some clinical trials of head and neck tumors [35]. The main mechanism by which CAP may exert its anti-tumor effects is through the generation of plasma-generated RONS, which can lead to oxidative stress in cancer cells [36]. The production of RONS in the plasma environment can have various effects on cancer cells, inducing cell death [37] and inhibiting proliferation [38]. In addition, CAP has been shown to have a number of other potential effects on cancer cells, including DNA damage [39,40], activating signaling pathways [9], and modulating immune responses [41].

**Figure 2:**
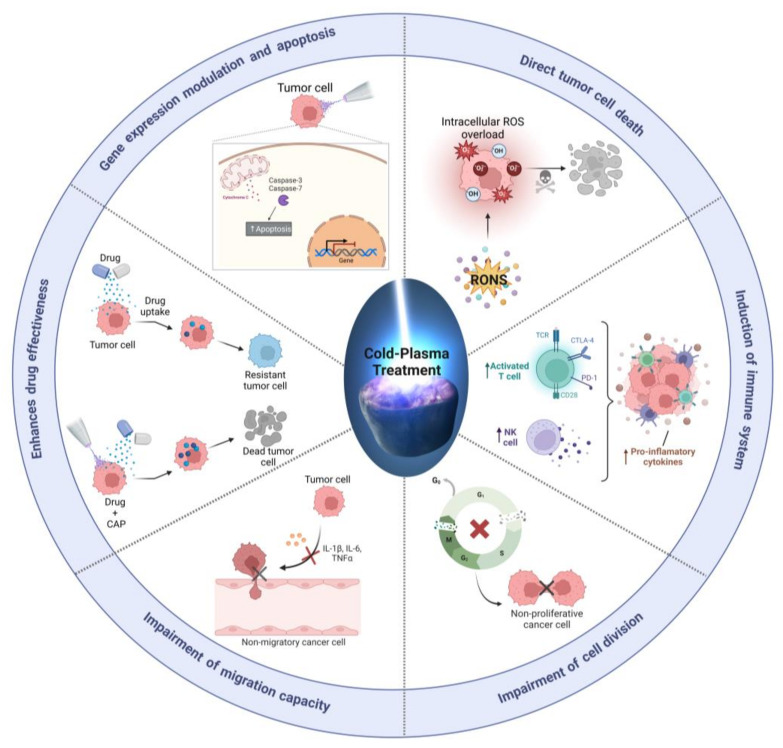
Schematic representation of the main mechanism reported to be induced by CAP in cancer. Briefly, external CAP-derived RONS penetrate the cell membrane by lipid peroxidation, then, intracellular ROS increases. Increase in intracellular ROS may induce DNA damage, impair cell division, migration, inducing the translocation of the cytochrome c and activation of apoptosis. The increase in RONS can also induce the activation of immune system to target tumor cells or enhance the effectiveness of chemotherapeutic drugs. Figure created using BioRender.com and DALL.E-2 (Accessed on 22 December 2022; openai.com/dall-e-2/).

The specific RONS that are formed during the cold plasma process can depend on a variety of factors, including the type of gas or liquid used, the plasma parameters, and the specific reactions that occur [16]. These RONS can appear through primary reactions. For example, cold plasma can ionize and dissociate gas molecules, resulting in the formation of atoms and radicals. When CAP is applied to a biological sample, usually covered by a liquid, the atoms and radicals can then react with other species in the gas phase or with the surfaces of the samples to produce secondary RONS. For example, atomic oxygen (O) and atomic nitrogen (N) can react with water vapor to form hydroxyl (OH) and nitrogen dioxide (NO_2_) radicals, respectively. These radicals can then react with other species to form more complex RONS, such as superoxide (O_2_^−^), hydrogen peroxide (H2O_2_), and peroxynitrite (ONOO^−^). In addition to these primary reactions, cold plasma can also generate RONS through secondary reactions, in which the plasma generates intermediate species that react with the gas or liquid to produce more RONS. For example, the nitrogen dioxide (NO_2_) radical can react with water to form nitrate (NO_3_^−^) and hydrogen peroxide (H_2_O_2_), while the hydroxyl (OH) radical can react with nitric oxide (NO) to form peroxynitrite (ONOO^−^) [42,43].In the liquid phase, cold plasma can generate RONS through a variety of mechanisms, including the direct interaction of the plasma with the liquid, the formation of excited species in the liquid, and the transfer of energy from the plasma to the liquid. The resulting RONS can have a range of effects on cancer cells (Figure 2). 

Plasma-generated RONS have been postulated as the main cytotoxic agents of CAP against cancer cells [37,44]. Plasma-generated RONS can have synergistic effects on cancer cells owing to their complexity [45]. For example, singlet oxygen produced by CAP can inactivate catalase, enabling the transport of H_2_O_2_ into cells and triggering cellular damage [37,46]. Other RONS produced by CAP, such as nitric oxide (NO), can disrupt cytochrome C oxidase, leading to increased levels of ROS and the induction of mitochondrial failure [37,46]. Thus, nitrites can react with H_2_O_2_ to produce hydroxyl peroxide (OHOO), which can cause lethal cell membrane peroxidation, DNA damage, and cell death [42,47].

However, the exact mechanism by which CAP induces cancer cell death is not completely understood (Figure 2). This is almost certainly because RONS are not targeted therapies, and each reactive species can affect multiple cellular signaling pathways as secondary messengers [48]. Besides, the concentration of RONS depends on diverse factors, such as the type of CAP device, the treatment time, the cell surface, the biochemical composition of the sample, and overall the protocol employed to deliver RONS into the tumor cells [9,16].

### 2.3. Plasma Oncology: Direct and Indirect Approaches

The delivery of RONS generated by plasma into tumors or cell cultures is an important aspect of its potential use as a therapeutic agent. Two distinct methods of plasma treatment have been described in the literature [9]: direct treatment and indirect treatment, also known as “two-step” or plasma-conditioned liquid (PCL) treatment (Figure 1C) [9,11].

In direct treatment, also known simply as CAP, the plasma beam is located directly above the surface of the sample, exposing the cells to all plasma components, including electromagnetic fields, UV light, ions, and RONS. In addition, de novo reactive species are generated by the plasma in contact with macromolecules and ions present in the liquid surrounding the biological sample.

In contrast, in the indirect treatment method, plasma never comes into direct contact with the biological samples. Instead, a liquid is treated with CAP, leading to the formation of new reactive species in the liquid. The plasma-conditioned liquid (PCL) is then transferred to a cell culture or injected into tumors in vivo as a delivery system for the plasma-generated RONS. In this process, living tissue or cell cultures are only exposed to RONS, ions, and stable active molecules that have been generated in the PCL but not to the remaining components of plasma.

The cocktail of RONS generated by either direct or indirect CAP application can lead to dysregulation at multiple cellular levels [47]. However, the determination of all RONS present in PCLs is complex, and the most frequently quantified long-lived RONS are H_2_O_2_ and NO_2_ [37,45,49], although it is known that a much more complex cocktail of RONS is formed in PCLs through diffusion from the gas phase to the liquid [9].

Some liquids that have been used in cold atmospheric plasma (CAP) treatments for cancer cells include the following:

- Water: is a commonly used liquid in CAP treatments for melanoma [50], breast and prostate [51] cancer cells in vitro, because of its low cost, availability, and the fact that it does not contain any ions or macromolecules that could potentially interfere with plasma-generated RONS.
- Ringers: a solution that is regularly used to maintain the physiological conditions of cells in culture. It contains ions and macromolecules that are similar to those found in the human body, which may be advantageous for in vitro applications in osteosarcoma [40,52] and for in vivo applications in prostate cancer [53].
- Phosphate-buffered saline (PBS): a liquid frequently used in biological and medical research. PBS is a buffer solution designed to maintain a constant pH and, as it contains ions and macromolecules that are similar to those found in the human body, it can support the survival and proliferation of cells while they are exposed to plasma-generated reactive oxygen and nitrogen species (RONS). Cytotoxicity of PCL using PBS as a liquid has been reported in glioblastoma [54] and breast cancer in vivo [55].
- Cell culture medium: To date, cell culture medium has been the most commonly employed liquid for studying the effects of plasma-generated RONS on cancer cells and in vivo tumors [11,44]. Noting that most of these studies have been conducted in monolayer cell cultures, with only a few studies conducted in vivo [44], it has been observed that the amount of RONS generated is greatly influenced by the biochemical composition of the cell culture medium sample, and slight differences in the composition, such as the inclusion of sodium pyruvate, can significantly alter the amount of RONS and the resulting cytotoxic effects [38]. While cell culture medium contains a variety of nutrients, hormones, and growth factors that can support the survival and proliferation of cancer cells, it is important to consider the effect of the biochemical composition of the medium on the generation of RONS [16] and their cytotoxic effects in order to accurately interpret the results of these studies.

Ultimately, the liquid of choice will depend on the specific goals and requirements of the study.

### 2.4. Cold Plasma for Cancer Treatment

Motaln et al. reviewed the diverse mechanisms by which direct CAP and PCLs induce cell death in tumor cells [56]. However, as stated before, it is difficult to propose a specific mechanism of cancer cell death by CAP-based therapies because RONS are not targeted therapies and each reactive species, ion, or charged particle can affect multiple cell signaling pathways as secondary messengers [57]. In addition, the concentration of RONS generated by CAP depends on various factors, such as the type of CAP device, treatment time, cell surface, well plate, volume of liquid, and biochemical composition of the sample [16]. Furthermore, the direct application of CAP jets used in biomedical applications can restrict the execution of basic molecular biology experiments, so researchers are often forced to use PCLs to study the signaling pathways affected by CAP [9]. This means that only the effects of long-lived RONS are considered, and therefore the results are not directly comparable to experiments using direct CAP treatment because the amount and nature of RONS will not be the same and other factors such as electromagnetic radiation and the remaining plasma components may affect cellular responses.

Nonetheless, there are several potential advantages to using CAP as a cancer treatment compared to other therapeutic options. Some of these advantages include the following:

- Non-toxic: direct CAP is a non-toxic treatment that does not generate harmful by-products or cause systemic side effects, unlike chemotherapeutic agents and other anticancer drugs, which can cause a wide range of side effects, including nausea, vomiting, hair loss, and immune suppression.
- Selectivity: CAP can selectively kill cancer cells while sparing normal cells. CAP generates reactive oxygen and nitrogen species (RONS) that preferentially damage DNA, proteins, and other biomolecules in cancer cells, leading to cell death. In contrast, many anti-tumor drugs are non-selective, killing both tumor and non-tumor cells, thus leading to the toxic side effects observed in patients.
- Versatile: Cold plasma can be directly applied to the tumor site, making it a potentially effective local treatment for solid tumors. Additionally, CAP can be delivered through numerous routes, including topical and intravenous administration, depending on the specific clinical setting. This versatility makes CAP a promising option for treating a wide range of cancer types and stages.
- Combinatorial: CAP can be used in combination with other anticancer drugs to enhance their efficacy and overcome drug resistance. Most likely because CAP can reverse the mechanisms that allow cancer cells to become resistant to drugs, thereby making them more sensitive to medication. In addition, CAP can stimulate the immune system to recognize and attack cancer cells, increasing the effectiveness of immunotherapies.

Overall, the use of CAP for cancer treatment offers many advantages over other therapeutic options, including its non-toxicity, selectivity, combinatorial potential, and versatility. Further research is needed to fully understand the mechanisms by which CAP eradicates cancer cells, ultimately contributing to developing a safe and adequate clinical therapeutic option using this technology.

## 3. Cold Plasma and Chemotherapy for Enhanced Cancer Treatment

Even though the mechanisms by which cold atmospheric plasma (CAP) may overcome drug resistance in tumor cells are not yet fully understood, there is some evidence to support its effectiveness in this regard. With this work, we aimed to review the scientific evidence supporting the use of CAP in combination with the most commonly used drugs in the clinic for cancer treatment (Table 1).

### 3.1. Temozolomide

Temozolomide (TMZ) is an imidazotetrazine derivative of the alkylating agent dacarbazine/alkylating agent, which was approved by the US Food and Drug Administration for the treatment of adult glioblastomas in 2005 [74,75]. This grade IV brain tumor, also called glioblastoma multiforme (GBM), is the most common and malignant form of primary astrocytoma occurring in the central nervous system (CNS), representing more than 60% of all brain tumors in adults [76,77]. It is characterized by aggressive behavior, high invasive potential, and resistance to current treatments, thus making it one of the most lethal cancers [76,77,78].

TMZ constitutes an effective first-line chemotherapeutic agent for the treatment of GBM when combined with radiation therapy and surgical resection. Over the past few years, the mechanism by which TMZ eliminates GBM tumors has been widely described. The cytotoxic effect of TMZ is mediated by the addition of methyl groups at the O6 and N7 positions on guanines and the N3 position on adenine, forming cytotoxic O6-methylguanine, N7-methylguanine, and N3-methyladenine. These cytotoxic bases form mismatched lethal base pairs during DNA replication, such as O6-methylguanine mispairing with thymine instead of cytosine, which results in double-strand breaks that induce cell cycle arrest at G2/M, ultimately leading to DNA damage and cell death [74,75,79].

Although the advances in surgical procedures, radiation therapy, and, more specifically, the use of TMZ and other alkylating agents have shown some improvement in the survival and quality of life of GBM patients, the treatment of this malignant brain tumor is still challenging and, in most cases, palliative, as the prognosis is still disheartening. Treatment failure for GBM has been attributed to the heterogeneous nature of these brain tumors and drug resistance, either acquired or intrinsic, due to drug efflux and DNA damage repair mechanisms, as well as the existence of hypoxic areas in the tumor and glioblastoma stem cells (GSC) [78]. 

The ability of GBM cells to activate epigenetic DNA repair mechanisms in response to TMZ constitutes a source of drug resistance, specifically through the expression of O6-methylguanine-DNA methyl transferase (MGMT), which reverses methylation of the O^6^ position of guanine and thereby repairs cytotoxic lethal base pairs, leading to the survival and resistance of tumor cells, instead of apoptosis [75,78,80]. Several studies found that TMZ-resistant GBM cells express higher levels of MGMT protein than TMZ-sensitive cells [75,81,82,83,84]. Moreover, MGMT promoter methylation, which results in the silencing of the enzyme (MGMT negative), has been associated with a favorable outcome, and patients may benefit from chemotherapy with the alkylating agent [78,85].

Furthermore, this chemotherapeutic drug has also been correlated with oxidative stress, which may play an important role in TMZ chemoresistance. Firstly, it was demonstrated that temozolomide treatment increased ROS production in GBM cells, resulting in the activation of AMP-kinase and cellular apoptosis [86], and more recently, it was observed that TMZ resistance in GBM cells was closely related to the antioxidant machinery system of these cells, most likely through the induction of the transcriptional factor NRF2 [87], a master regulator of the antioxidant response, and an enhancement of the synthesis and utilization of glutathione (GSH) [88] by resistant tumor cells after TMZ treatment.

In this regard, CAP used in combination with TMZ treatment has been found to inhibit cell growth in a dose-dependent manner, inducing DNA damage in vitro in human GBM cell lines both sensitive (U87MG and LN229, MGMT negative) and resistant (LN18, MGMT positive) to TMZ [58,59]. In addition, Koritzer et al. observed that CAP treatment not only inhibited cell proliferation but also reduced clonogenicity and led to a significant cell cycle arrest in the G2/M phase [58]. Interestingly, Gjika et al. reported that the combination of both treatments successfully reduced cell migration and increased the expression of surface integrins αvβ3 and αvβ5 in GBM cell lines [59].

These studies performed by Koritzer et al. and Gjika et al. were the first to introduce CAP as a promising therapeutic option for sensitizing cells to TMZ treatment in monolayer cell cultures of glioma cells, independently of their MGMT status. In line with these results, Soni et al. [60] corroborated that TMZ anti-tumor activity can be enhanced by co-treatment with direct CAP not only in vitro but also in vivo, as the combination treatment of CAP + TMZ significantly inhibited the viability of all GBM cell lines assayed (TMZ-sensitive, A172 and U87MG, and TMZ-resistant, T98G) and a single non-invasive CAP application was enough to potentially sensitize intracranial tumors in mice to subsequent low-dose TMZ therapy; therefore, preventing GBM progression. 

As previously mentioned, the administration of plasma-generated RONS by PCLs seems to be a feasible option to treat inner tumors. Using PBS as a carrier liquid of RONS, Shaw et al. observed that PCL enhanced the efficacy of TMZ towards both TMZ-sensitive and TMZ-resistant cell lines in vitro, in the same way as it was previously described for direct CAP combined with TMZ [61]. Notably, co-treatment of TMZ with direct CAP has a greater cytotoxic effect in TMZ-sensitive and TMZ-resistant GBM 3D spheroid models than the combination of PCL and the alkylating agent. It should be noted that 3D spheroids were more resistant to treatments than cells in monolayers, and CAP was more efficient than PCL in eliminating the cell viability of 3D glioblastoma spheroids [61].

Overall, it has been described that CAP synergistically enhances the therapeutic effect of TMZ in 2D and 3D sensitive and resistant models and restores GBM cell lines’ sensitivity to the alkylating agent. However, the mechanism by which CAP restores glioma cells’ sensitivity to temozolomide remains unknown. It is assumed that the exogenous source of reactive oxygen and nitrogen species (RONS) generated by CAP and TMZ results in an imbalance in the oxidative homeostasis and antioxidant machinery that increases the intracellular ROS levels, which seemingly decreases GSH levels and the expression of the antioxidant enzyme GPX4, thus amplifying DNA damage and eventually causing oxidative stress-mediated cell death [61]. 

Although the synergistic application of CAP and TMZ seems to be a promising option to induce cell cycle arrest and apoptosis in contrast to individual therapies, it remains unclear whether combined CAP–TMZ therapy will be effective in treating glioblastoma patients. Further investigation is paramount to clarify how CAP could affect the changes in MGMT promoter methylation after its exposure. 

### 3.2. Doxorubicin

Doxorubicin (DOX) is a natural anthracycline isolated from a mutant strain of *Streptomyces peucetitus* that has antibiotic and chemotherapeutic effects by blocking topoisomerase II (TOP2A), resulting in DNA damage-associated cell death in cancer cells [89]. DOX is an extensively used chemotherapeutic agent that was approved by the FDA in the 70s. Since its approval, DOX has become a first-line chemotherapy drug for many tumors, such as sarcomas, acute lymphoblastic/myeloblastic leukemia, neuroblastoma, breast, small cell lung, ovarian, bladder, gastric, thyroid, Wilms tumor, Hodgkin’s, and cutaneous T-cell lymphoma [90]. 

The main problem associated with DOX is its high cytotoxicity. When administered systemically, owing to its ability to block DNA replication, it induces several side effects, such as nausea, vomiting, alopecia, and dangerous cardiotoxicity [91,92,93]. In addition to these side effects, one of the most critical limitations of the use of DOX is the appearance of resistant clones. Although the most widely described mechanism of DOX resistance is the regulation of the expression and activity of the drug efflux pump ABCB1 (also known as MDR1 or P-gp) [94,95], other mechanisms have also been described, such as miRNAs [96,97], regulation of DOX targets such as TOP2A or P53 [91], or the regulation of stemness-related factors [98,99]. The resistant phenotype induced by DOX has encouraged researchers to explore mechanisms to overcome DOX resistance, including the use of nanocarriers [100], novel approaches able to eliminate drug efflux ABC pumps [95], and compounds or methodologies that take advantage of the altered pathways of resistant cells, such as increased sensitivity to ROS apoptosis or bioenergy states (e.g., inhibition of glycolysis using hexokinase II inhibitors).

DOX induction of oxidative stress in cancer cells, has recently led to the proposal of CAP as a secondary source of RONS, which in combination with DOX may overwhelm the antioxidant defenses of tumor cells. Sagwal et al. demonstrated that the administration of direct plasma together with either free or nano-encapsulated DOX exerted a strong cytotoxic synergistic effect in murine and human melanoma cells by accelerating the apoptotic effect of DOX. Mechanistically, the combination of CAP and DOX promotes the upregulation of the organic cationic transporter SLC22A16, resulting in higher intracellular doxorubicin accumulation [62,63,64]. Another study demonstrated that, on the one hand, a low dose of PCL increased intracellular ROS, whereas, on the other hand, a higher dose of PCL enhanced the cytotoxic effects of DOX in breast cancer cell lines. Interestingly, the intracellular ROS levels in the combinatory treatments were significantly higher than those generated by DOX or PCL treatment alone [65].

Noteworthy, all of these studies agreed that the accumulation of excessive oxidative stress could be a promising option to treat tumors. However, most of the studies were performed using monolayer cultures, which overlook the potential harmful effects of RONS in stromal cells or the presence of a 3D environment, which often limits the diffusion of ROS into the tumor, emphasizing the need to properly characterize the outcome of DOX and CAP combinatorial therapy in order to elucidate whether it could be described as a prospective therapy. 

Recently, Mateu-Sanz et al. showed that the use of PCL improved the cytotoxic effect of DOX in a 3D-engineered model of prostate cancer. Prostate cancer often metastasizes in bone; therefore, after seeding them into a bone-like scaffold, prostate cancer cells were treated with PCL. The administration of indirect plasma was able to enhance the cytotoxic potential of DOX as the combination of PCL and DOX resulted in the downregulation of redox defenses (CAT1, SOD2, and GPX1), induced apoptosis, and downregulated the expression of well-studied drug efflux ABC pumps (ABCC1, ABCB1, and ABCG2) associated with DOX resistance. The combination of PCL and DOX resulted in an enhanced uptake of DOX coupled with an overload of intracellular ROS in prostate cancer cells [39]. Moreover, the authors observed that PCL improves the migratory and clonogenic potential of prostate cancer cells in a monolayer. Notably, this study highlighted the fact that PCL and DOX did not affect the viability of healthy stem cells or human osteoblasts [39].

Despite these promising results, some questions remain unanswered, such as how CAP or PCLs could eliminate DOX-resistant cell models or whether the combination of CAP and DOX affects tumor growth and/or healthy tissues in vivo. Overall, these factors must be elucidated prior to considering CAP and DOX co-treatment as a promising anti-cancer therapy. 

### 3.3. Epirubicin

Similar to DOX, epirubicin [101] is another anthracycline that causes DNA damage and induces cell death by targeting and inhibiting topoisomerase-II activity, a key enzyme in the maintenance of chromosomal topological status during DNA replication, which leads to DNA double-strand breaks [102,103]. This drug is commonly used for the treatment of several malignancies, including breast [104], gastric [105], non-small cell lung cancer [106], and colorectal cancer [103]. Together, DOX and EPI constitute the cornerstone of chemotherapy for the management of early breast cancer, since they confer higher survival rates compared to other non-anthracycline drugs [107]. 

Despite its widespread use, especially in breast cancer, in clinical practice, EPI administration is compromised by its associated cardiotoxicity effects and subsequent heart failure, especially in patients with breast and hematopoietic cancer such as lymphoma. It has been shown that childhood cancer survivors have a 5–15-fold increase in risk for congestive heart failure (CHF) compared to the general population [107,108,109], and the risk for cardiac dysfunction correlates with increasing anthracycline doses, causing permanent damage at the cellular level [110]. The disease starts at the subcellular level when there is myocellular injury (apoptosis, necrosis, DNA damage, etc.) that may be reversible, followed by subclinical injuries such as left ventricle [111] systolic dysfunction, which eventually induces LV remodeling, leading to chronic and irreversible chemotherapy-induced cardiomyopathy [112]. 

Other features, such as the identification of biomarkers that may confer higher resistance to epirubicin-based treatments [113,114,115], have highlighted the need to seek new strategies for dispensing this drug. In this regard, a synergistic cytotoxic effect has been reported when EPI is combined with CAP. It has been described that cold plasma exposure increases toxic effects in melanoma cells pretreated with EPI [62], as well as that it significantly induces caspase 3/7 activity when compared with monotherapy. These results were also observed in melanoma 3D cultures, in which tumor spheroids pretreated with epirubicin (1 µM) showed enhanced toxicity upon combination with plasma oxidants. The combination treatment resulted in an increased DNA damage response, as evidenced by the increased phosphorylation of ATM, formation of gamma-H2AX foci, and formation of micronuclei. In addition, the secretion of the immunogenic cell death markers ATP and CXCL10 was enhanced in the cell culture supernatants following combination treatment. The observed synergistic effects in tumor cells were likely due to the upregulation of the organic cationic transporter SLC22A16 by plasma treatment, leading to an increased intracellular accumulation of DOX and EPI [62]. 

### 3.4. Cisplatin

Cisplatin (CIS) is an alkylating drug formed as a result of the electrolysis of platinum electrodes, with a chemotherapeutic effect by causing adducts in the DNA [116]. As with DOX, CIS is one of the most commonly used anticancer drugs for the treatment of many solid tumors, such as sarcoma, head and neck, gastric, bladder, lung, cervical, breast, leukemia, testicular, and ovarian cancers [117]. The most common side effect of continuous CIS treatment is nephrotoxicity [118,119,120], although ototoxicity, cardiotoxicity, neurotoxicity, and hepatotoxicity have also been reported [116,117].

Decreased drug uptake by upregulating organic cation transporters such as CTR1, increased sequestration of cisplatin by GSH, an improvement of the DNA repair mechanism and DNA damage tolerance, or the genetic and epigenetic regulation of several apoptosis and oxidative stress factors have been reported as mechanisms of cisplatin resistance [121,122]. Thus, several studies have demonstrated ways to overcome CIS resistance: the use of nanocarriers [123], phytochemicals [124], or STAT3 inhibitors [125].

Given the close relationship between CIS treatment and the production of reactive oxygen and nitrogen species, a possible synergistic effect between CAP and CIS should be considered. Similar to the promising results observed with the combination of PCL and DOX, combined treatment with direct CAP and CIS demonstrated a strong synergistic effect in head and neck cancer without affecting healthy fibroblastic cells [66,67]. CIS treatment decreased the viability of both oral squamous cell carcinoma cell lines and healthy human fibroblasts in a dose-dependent manner; however, the combination of CAP with the alkylating drug provoked a more efficient decrease in tumor cell viability, maintaining the viability of fibroblasts. Particularly, this study showed that it is feasible to reduce CAP exposure time and CIS concentration and still achieve synergistic effects that eliminate cancer cells without affecting their healthy counterparts. In line with this data, Li et al. tested the combination of CIS and CAP in hepatocellular carcinoma (HCC) cells, demonstrating that direct CAP treatment largely eliminates stem-like properties in HCC cells, often related to greater chemo-resistance, while recording only a marginal decrease in healthy cell viability. The researchers also examined the efficacy of the combined application of PCL and CIS, showing increased induction of cell death in Huh7 cells, while the combination of PCL with other drugs such as sorafenib, doxorubicin, or trametinib was less effective. These results suggest that PCL increases the effect of CIS in HCC by selectively inducing cell death in HCC subpopulations with stem-like properties, but not in healthy liver cells [68]. To date, the combinatory effect of CAP or PCL with CIS has been entirely studied in monolayer cultures; therefore, it is indispensable to intensify these findings using more complex in vitro models and overall, elucidate the molecular mechanisms associated with the combinatory synergistic and/or additive effect of plasma and CIS to develop effective therapies.

### 3.5. Paclitaxel

Paclitaxel (PTX), also known as Taxol, is a plant alkaloid naturally produced in the bark and needles of Taxus brevifolia, and it is one of the most commonly used anticancer drugs, being applied for the treatment of several types of cancer, including breast, ovarian, lung, esophageal, or Kaposi’s sarcoma, among others [126,127]. PTX exerts its anti-tumor activity by arresting cells in metaphase during mitosis due to its ability to bind to the polymeric form of tubulin. This binding stabilizes microtubules and prevents their dissociation, which induces cell cycle arrest at the G2/M transition [128,129,130]. 

Platinum-based chemotherapy drugs, such as paclitaxel, are commonly used to treat various types of cancer. However, some cancer cells can develop resistance to PTX, and although the exact molecular mechanisms that contribute to paclitaxel resistance are not fully understood, several factors have been identified as potential contributors.

One mechanism that has been studied is the increased expression of certain proteins, such as P-glycoprotein and multidrug resistance-associated protein, which can pump paclitaxel out of cancer cells, making it less effective. Additionally, some cancer cells may have mutations in genes that are targeted by paclitaxel, which can also lead to resistance. Thus, cancer cells acquire PTX resistance through the upregulation of xenobiotic transporters, such as ABCB1 and MRP3, or through the expression of alternative tubulin isoforms [131,132]

Another mechanism that has been proposed to be responsible for PTX resistance is the activation of certain signaling pathways, such as the PI3K/Akt/mTOR pathway, which can protect cancer cells from the effects of the drug [133]. In addition, changes in the structure of microtubules in cancer cells can contribute to resistance [134]. One potential mechanism by which CAP can restore paclitaxel sensitivity in cancer cells has been described by Mihai et al.; here, PCL was administered in combination with PTX in breast cancer cell lines, showing that plasma-generated RONS increased the cytotoxic effects of PTX against monolayer and 3D spheroids of breast cancer cells, and that the combination acted synergistically to eliminate the clonogenic and migratory potential of breast cancer cells [69]. Additionally, direct CAP application in vitro restored the sensitivity of breast cancer cells to PTX by reducing the expression of drug-resistance genes, suggesting that CAP can make PTX-resistant cells more sensitive to paclitaxel and other chemotherapy drugs. So, it is clear that the combination of PTX and plasma-based therapies can trigger apoptosis, or programmed cell death, which can make breast cancer cells more sensitive to PTX by modulating their gene expression pattern related to PTX resistance [70]. 

### 3.6. Tamoxifen

Tamoxifen was first synthesized as a contraceptive pill in 1962. However, the compound was found to stimulate rather than suppress ovulation in women. In an attempt to not withdraw the drug from the market, it was repurposed to treat breast cancer [135]. It has been used not only as a treatment but also as a chemopreventive drug, as it blocks the effects of estrogen on breast tissue. In breast cancer cells, tamoxifen can slow or stop tumor growth by blocking estrogen receptors on cancer cells. However, some cancer cells can become resistant to the effects of tamoxifen, which means that the medication is no longer effective in stopping tumor progression. This can occur when cancer cells develop mutations that allow them to continue growing, even in the presence of tamoxifen. These mutations can affect estrogen receptors on cancer cells, making them less responsive to medication. Additionally, some cancer cells may develop mechanisms that allow them to bypass the effects of tamoxifen, such as increasing their production of enzymes that break down the medication.

Several mechanisms may contribute to tamoxifen resistance in breast cancer cells [136]. Some of these mechanisms include: (I) Mutations in the estrogen receptor (ER) gene, which encode a protein found on the surface of breast cancer cells. Tamoxifen binds to the estrogen receptor and blocks its activity. However, some breast cancer cells can develop mutations in the ER gene that alter the structure of the estrogen receptor protein, making it less responsive to tamoxifen. (II) Increased production of enzymes that break down tamoxifen: Some breast cancer cells can increase their production of enzymes that break down tamoxifen, such as cytochrome P450 enzymes. This can reduce the effectiveness of the medication by decreasing its concentration within the cancer cells. (III) Activation of alternative signaling pathways. (IV) Changes in the tumor microenvironment: Some breast cancer cell subpopulations can alter the tumor microenvironment in ways that promote tamoxifen resistance, such as by recruiting immune cells that support tumor growth or by secreting growth factors that stimulate cancer cell proliferation.

CAP has been shown to directly kill tamoxifen-resistant breast cancer cells. Direct CAP treatment has been found to modulate the expression of 18 genes, including MX1 and HOCX6, which are involved in the development of tamoxifen resistance. After CAP treatment, the expression of these genes was restored to the levels observed in normal MCF7 cells, at both the mRNA and protein levels. This suggests that CAP may be able to reverse tamoxifen resistance by restoring the sensitivity of cancer cells to tamoxifen [65,71]. 

### 3.7. 5-Fluorouracil

5-fluorouracil (5-FU) is a chemotherapy agent used in the treatment of various types of cancer, including colon, rectal, breast, stomach, and pancreatic cancer. This drug functions by inhibiting DNA synthesis, a crucial process for the growth and division of cancer cells. 5-FU is typically administered intravenously or as a topical cream or solution [137]. However, like many other anti-tumor drugs, its use is limited by its side effects, such as nausea, vomiting, diarrhea, mouth sores, and a low white blood cell count. It is often administered in conjunction with other chemotherapeutic agents or radiation therapies. In addition to the side effects it causes, 5-fluorouracil resistance has been reported [138,139,140]. Several mechanisms may contribute to drug resistance in cancer cells treated with 5-FU. In this regard, it has been described that some cancer cells may produce high levels of two enzymes known as dihydropyrimidine dehydrogenase (DPD) and thymidylate synthase (TS), which can metabolize 5-FU and reduce its effectiveness. Cancer cells may also develop mutations in the TS gene, which can decrease the sensitivity of cells to 5-FU. Notably, tumor cells can eventually acquire multidrug resistance genes and stem cell properties that contribute to drug resistance. In the search for different strategies to tackle 5-FU resistance, recent studies have demonstrated that the use of DBD devices can decrease the viability of 5-FU-resistant human hepatocarcinoma cells [72], and the application of a plasma jet has been found to increase the effectiveness of the drug in vitro on cholangiocarcinoma cells [73]. However, the exact mechanisms underlying these effects are not yet fully understood.

Altogether, these studies corroborated the relevance of developing new therapeutic strategies such as CAP, as well as the need for further research to determine whether the combination of anticancer drugs and CAP can be beneficial for patients. This requires the improvement of preclinical research using 3D and in vivo models, which is currently a pending topic.

## 4. Challenges in Proposing CAP Therapy for Cancer

CAP is a technology in continuous development and expansion. However, despite the advances achieved in this field, which have allowed us to expand our knowledge about the possible therapeutic uses of plasma and how it could enhance the effects of other anti-tumor drugs, it is paramount to continue our work, broadening the different lines of research. 

### 4.1. A Closer Look at CAP Selectivity in Targeting Cancer Cells

It is believed that the cocktail of reactive oxygen and nitrogen species (RONS) generated by cold atmospheric plasma (CAP) can be used to target cancer cells without significantly affecting non-malignant cells and surrounding tissues [141,142]. The concentration and type of RONS generated by CAP heavily depend on plasma application parameters, such as distance and treatment time, as well as on the chemical/biochemical composition of the liquid [16]. However, only a few studies have directly compared the effects of CAP on tumor and non-tumor cells under the same experimental conditions.

The specific vulnerabilities of cancer cells to CAP have been reported in many studies, which have shown that this sensitivity mainly depends on the accumulation of intracellular ROS, most likely because cancer cells, unlike healthy cells, have less cell membrane cholesterol [143] and a higher number of aquaporins in the cell membrane [142], among other possible factors. Interestingly, some researchers have proposed that cancer cells are more sensitive to CAP than non-tumor cells because tumors rely on a different redox system in response to plasma-generated RONS such as peroxynitrite, superoxide, and hydrogen peroxide [42,144,145,146]. Accordingly, this model of cancer cell susceptibility suggests that CAP primarily affects tumor cells, as they are more likely to surpass the threshold of oxidative stress toxicity. Hence, it is possible that a therapy based on oxidative stress could be selectively effective against cancer cells. Oxidative stress, described as an imbalance between the production of reactive oxygen species and the body’s ability to detoxify them, has been shown to play a decisive role in the development and progression of cancer [48,57]. In 2009, Trachootham et al. proposed that cancer cells may be more susceptible to oxidative stress than normal cells, and that targeting this vulnerability could become a potential therapeutic strategy [147]. 

Nowadays, in the era of personalized medicine and next-generation sequencing for cancer treatments, the use of an oxidative stress-based therapy such as cold plasma could be controversial [57,148], compounded by the lack of studies comparing the effects of CAP on healthy and cancer cells under the same experimental conditions. Tornin et al. showed that incorrect use of plasma cell therapy can similarly eliminate both cancer stem cells and healthy stem cells [38]. In addition, these authors recently concluded that a higher amount of reactive oxygen and nitrogen species (RONS) is necessary to completely eliminate cancer cells in 3D models such as 3D-engineered cultures [52] and organotypic cultures [40], which could have serious negative effects on healthy cells [38,40,52]

Additionally, some studies have shown that improper use of CAP can cause ulceration and necrosis [149], and high doses of RONS produced by CAP can have unfavorable effects on red blood cells [150]. The novelty of the field and the large number of devices and methodologies in use pose a challenge when comparing results and studying the potential of CAP as a selective anti-tumor agent.

### 4.2. CSC: A Pending Subject of Study

Cancer stem cells (CSCs) are a subpopulation of cancer cells responsible for tumor growth, maintenance, and recurrence [151,152]. CSCs resemble normal stem cells in that they have the ability to self-renew and differentiate into multiple cell types. Unfortunately, CSCs are also capable of unlimited proliferation and are resistant to the damaging effects of chemotherapy and radiation therapy [98].

CSCs display several properties that make them a major source of drug resistance in cancer, as they have a unique cellular and molecular structure that renders them resistant to the effects of chemotherapy and radiation therapy. These include: (I) enhanced antioxidant machinery due to high levels of antioxidant enzymes such as superoxide dismutase and catalase, and the ability to upregulate the expression of genes encoding these enzymes, further enhancing their resistance to ROS [153,154]; (II) augmented DNA repair capacity, high levels of DNA repair enzymes can quickly restore DNA breaks and other damages caused by chemotherapy and radiotherapy; (III) self-renewal capacity that leads to the production of more cancer stem cells, contributing to tumor expansion and the development of drug resistance.

ROS are generated as a byproduct of normal cellular metabolism; however, they can also be produced by chemotherapy and radiotherapy. Notably, it has been reported that ROS can also increase the survival of cancer stem cells (CSCs) through different mechanisms [153,154,155].

1. Antioxidant enzymes: high levels of antioxidant enzymes observed in CSCs protect them from ROS-induced DNA damage. These enzymes catalyze the conversion of ROS into less harmful molecules such as hydrogen peroxide and water, which reduces their cytotoxic effects. Enhanced expression of scavenging genes, including superoxide dismutase, glutathione peroxidase, and catalase, helps maintain intracellular ROS at nearly identical levels to those of normal stem cells. Besides, the activation of oncogenic transcription factors, such as c-Myc, can increase ROS levels and activate NRF2, a transcription factor that upregulates genes involved in detoxification and antioxidant activity. NRF2 activates the expression of efflux transporters and anti-apoptotic proteins, and contributes to iron homeostasis, making it a potential target for CSCs therapy [156].
2. Upregulation of antioxidant genes: CSCs can upregulate the expression of genes that encode antioxidant enzymes, such as GPXs, in response to ROS. In addition, CSCs are characterized by the elevated expression of the cellular markers CD44 and ALDH, which are associated with enhanced GSH synthesis and stronger protection against ROS [157]. The ALDH enzyme, highly expressed in CSCs [158], has a detoxifying function by reducing ROS and generating antioxidant compounds, therefore protecting against alkylating agents and increasing the activation of DNA repair mechanisms.
3. Activation of survival pathways: ROS stimulate the expression of anti-apoptotic proteins, such as Bcl-2, which inhibits the initiation of programmed cell death, and promote the activation of survival pathways such as PI3K/AKT/mTOR or STAT3 [57]. For instance, it has been reported that activation of DNA damage responses increases the number of CSCs by approximately 2–4 fold [8]. In glioma, activation of DNA damage checkpoints was found to be more effective in CD133+ cells after radiation exposure than in their CD133- counterparts [15]. Enhanced DNA repair mechanisms present in glioblastoma stem cells, compared to progenitor cells [128], make these cells highly sensitive to the inhibition of PARP and ATR [129]. Furthermore, overexpression of polymerase η confers resistance to cisplatin in ovarian cancer cells, whereas activation of mir-93, which regulates polymerase η expression, increases the sensitivity of CSCs to cisplatin [130]. Nevertheless, it should be noted that the response to DNA damage can be a double-edged sword and have opposite effects. Whereas in non-tumor stem cells, this process promotes optimal functioning of healthy tissues, in cancer stem cells it leads to survival and resistance. CSCs can tolerate high levels of replication stress through this mechanism. In fact, CSCs can resist chemotherapeutics specifically designed to damage DNA [129].

Nonetheless, the effects of plasma-generated RONS on CSC are not well understood, and the available research on this topic is conflicting. Some studies have suggested that CAP can eliminate the stem-like properties of CSCs and reduce their ability to regenerate tumors. For instance, PCLs have been shown to reduce the expression of stem cell markers, such as ALDH1 [159], and inhibit CSC-like properties in combination with cisplatin [160]. On the contrary, other studies have suggested that PCLs enhance the survival and proliferation of CSCs in osteosarcoma [52]. In this regard, CAP has been shown to upregulate the expression of the GPX1 antioxidant enzyme [39], which protects CSCs from ROS-induced DNA damage. In addition, CAP has been shown to activate signaling pathways that promote the survival and proliferation of CSCs, such as the Akt-mTOR pathway [161].

Therefore, it is challenging to use ROS to eliminate cancer stem cells (CSCs), as it is imperative to first determine whether CAP is capable of eliminating CSCs or enhancing their survival. It is worth noting that CAP and PCL have been extensively studied for their ability to increase stem cell properties in healthy cells and tissues [34], and given their similarity to cancer stem cells, it is possible that CAP and PCL may also promote CSC properties. However, it is also conceivable that the effects of CAP on CSCs may be context-dependent, depending on the specific tumor type, stage, and treatment regimen.

## 5. Conclusions and Future Perspectives

Cold atmospheric plasma (CAP) is a promising therapy that utilizes the generation of reactive oxygen and nitrogen species (RONS) to target cancer cells. Although plasma has shown potential in overcoming drug resistance in cancer cells, the mechanisms by which this is achieved are yet to be completely elucidated. This review has attempted to summarize the potential mechanisms of action of CAP in the treatment of cancer. 

One of the most outstanding mechanisms is the direct killing of cancer cells. RONS generated by CAP can damage DNA, proteins, and other biomolecules in cancer cells, leading to cell death, including cancer cells that are resistant to drugs, potentially reducing the tumor size. Another potential mechanism is the induction of anti-tumor immunity, as CAP has been shown to stimulate the immune system to recognize and attack cancer cells, thereby enhancing the effectiveness of some drugs. In this regard, CAP has been shown to increase the infiltration of immune cells into the tumor, enhance the expression of immune checkpoint molecules, and stimulate the production of pro-inflammatory cytokines that promote anti-tumor immune responses. (Figure 2). 

**Figure 3:**
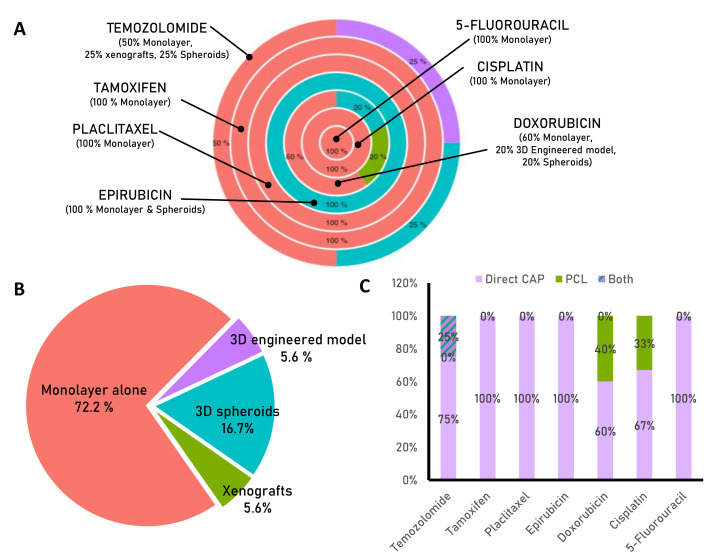
Statistics of studies related to CAP combined with pharmaceuticals. (**A**) The pie chart shows the percentage of trials conducted in monolayer, 3D models, or in vivo for each drug indicated. (**B**) The pie chart indicates the percentage of studies conducted in vitro using monolayer cultures (72.2%), and less than 6% using xenografts or 3D-engineered models. (**C**) The bar chart shows the percentage of studies that employed direct or indirect application of CAP for each drug. Most of the experiments in the literature have used CAP treatments combined with drugs, mainly in monolayer cultures.

Nonetheless, one of the major drawbacks of this novel technology is that despite the promising results of CAP to improve the effectiveness of conventional drugs, the vast majority of experiments were performed using monolayer cell cultures (Figure 3A,B) which may not accurately reflect the complex and heterogeneous nature of the in vivo tumor microenvironment, the side effects, or the presence of CSCs within the tumor. Furthermore, in vitro studies do not follow common application criteria, many different experimental setups are implemented, and even small differences—apparently—such as the cell culture media [38], the plasma device employed, or the application of CAP or PCLs (Figure 3C), can lead to huge differences and misleading conclusions.

Therefore, there is an urgent need to improve scientific research in this area by incorporating 3D models and studying the role of CSCs and the tumor microenvironment in CAP therapy.

There are several aspects to consider and be aware of before finally proposing cold atmospheric plasma (CAP) as a prospective therapy against drug resistance in cancer. These include:

1. Plasma mechanism of action: It is important to understand how CAP exerts its effects on cancer cells and its underlying mechanisms of action. This involves studying the role of reactive oxygen and nitrogen species (RONS) and other plasma-generated species as well as the impact of CAP on signaling pathways, gene expression, and cell cycle regulation.
2. Effect of CAP on cancer stem cells: Cancer stem cells (CSCs) are a subset of cancer cells resistant to chemotherapy and radiotherapy, and they are thought to play a key role in the development and progression of cancer. It is important to study the effect of CAP on CSCs to determine whether this technology can be proposed as a potential therapeutic agent against drug resistance.
3. Existence of the tumor microenvironment: It is also significant to study the impact of CAP on the tumor microenvironment, which includes the surrounding cells, extracellular matrix, and soluble factors, and plays a crucial role in the growth and survival of cancer cells and CSCs. The contribution of plasma to eliminating tumor surroundings could support its proposal as a therapeutic strategy against drug resistance.
4. Preclinical studies: Prior to considering CAP as an anticancer therapy, it is essential to conduct preclinical studies using appropriate model systems, such as three-dimensional (3D) organoids, engineered models, or spheroids, to better mimic the in vivo situation and understand the effectiveness of CAP.
5. Clinical trials: The effectiveness of CAP as a therapy against drug resistance in cancer will have to be evaluated in clinical trials involving human subjects. In this regard, carefully designing and conducting these trials will accurately assess the safety and effectiveness of CAP in this setting.

To overcome these challenges and thoroughly exploit CAP as a potential strategy for cancer treatment, it is necessary to examine its mechanisms of action and explore potential combinations with clinical drugs. Combinatorial therapy with CAP and chemotherapy drugs may be a priority in this field, as it may help eliminate cancer and minimize side effects more effectively. Ultimately, the objective is to use CAP as part of a personalized approach to treating cancer, considering the unique characteristics of each patient’s tumor and its potential interactions with other therapies. The identification and management of potential inconveniences, such as the effects on tumor heterogeneity and the control of excess ROS in healthy counterparts, will be crucial in maximizing the safety of proposing cold plasma as a prospective therapy. Overall, the use of cold atmospheric plasma as a cancer treatment is a promising area of research; however, further studies are required to fully evaluate its potential benefits and drawbacks.

## Figures and Tables

**Table 1:** List of chemotherapeutic agents studied in combination with CAP or PCLs.

**Drug**	Cancer Type	Biological Model	Cold-Plasma Application	Type of Plasma Device	Main Results Obtained and Reference
Temozolomide	Human Glioblastoma	Monolayer	Direct CAP	Dielectric barrier discharge (DBD)	CAP treatment can reestablish the sensitivity of resistant glioma cells to temozolomide [58]
	Human Glioblastoma	Monolayer	Direct CAP	Plasma Jet	CAP amplifies cytotoxicity of Temozolomide, causes cell cycle arrest and DNA damage and inhibits cell migration [59].
	Human Glioblastoma	Monolayer/Xenograft	Direct CAP	Plasma Jet	CAP increase the anti-tumoral activity of temozolomide in vitro and in vivo [60].
	Human Glioblastoma	Monolayer/spheroids	Direct CAP + PCLs (PBS as liquid)	Plasma Jet	CAP and PCL are effective treatments against temozolomide resistant cells. Only CAP combined with temozolomide is effective in eliminating 3D spheroids [61].
Doxorubicin	Murine Melanoma	Monolayer/spheroids	Direct CAP	Plasma Jet	Direct CAP application increase the uptake of DOX and sinergisitically eliminates cell viability. CAP activates inmune system cells [62].
	Murine and human melanoma cells	Monolayer	Direct CAP	Plasma Jet	CAP increases the efectiveness of doxorubicin and supports the cancer-selective cytotoxic effect of liposomal nanoparticles loaded with doxorubicin [63].
	Human Melanoma cells	Monolayer	Direct CAP	Plasma Jet	Continuous doxorubicin treatment followed by CAP treatment is more effective than simultaneous drug and plasma treatment [64].
	Human Breast cancer	Monolayer	Indirect PCLs (cell culture media as liquid)	Plasma Jet	Sinergistally elimination of cell viabilityby the combination of PCL and doxorubicin [65].
	Human metastatic bone, prostate cancer	Monolayer/engineered model	Indirect PCLs (cell culture media as liquid)	Plasma Jet	PCL loss cytotoxicty in 3D environments. Combination of low dose of PCL and DOX improves cytotoxic effects of doxorubicin unafeccting healthy counteraparts [39].
Epirubicin	Murine Melanoma	Monolayer/spheroids	Direct CAP	Plasma Jet	Direct CAP application increase the cytotoxic effects of epirubicin an other chemoterapeutic agents [62].
Cisplatin	Human head and neck squamous carcinoma	Monolayer	Direct CAP	Dielectric barrier discharge (DBD)	Direct CAP increase the efectiveness of low dose of CIS [66]
	Human oral squamous carcinoma	Monolayer	Direct CAP	Plasma Jet	The combination of CAP and cisplatin has a synergistic anti-cancer effect, although normal fibroblast cells are less sensitive to the combinatory treatment [67].
	Hepatocellular Carcinoma	Monolayer	Direct CAP + PCLs (cell culture media as liquid)	Dielectric barrier discharge (DBD)	PCL in combination with cisplatin has an additive cytotoxic effect that is higher than when PCL is combined with other drugs (sorafenib, doxorubicin, or trametinib). CAP targets cancer stem cell properties and has less impact on healthy cells [68].
Paclitaxel	Human Breast cancer	Monolayer	Indirect PCLs (cell culture media as liquid)	Dielectric barrier discharge (DBD)	PCLs increase the cytotoxic potential of paclitaxel [69].
	Human Breast cancer	Monolayer	Direct CAP	Dielectric barrier discharge (DBD)	CAP restores the paclitaxel sentitive status [70].
Tamoxifen	Human Breast cancer	Monolayer	Direct CAP	Dielectric barrier discharge (DBD)	CAP restores the paclitaxel sentitive status by modulating gene expression related to drug resistance [71].
5-Fluorouracil	Human Hepatocarcinoma	Monolayer	Direct CAP	Dielectric barrier discharge (DBD)	Decrease in cell viability of 5-Fluorouracil resistant cells [72].
	Human cholangio-carcinoma	Monolayer	Direct CAP	Plasma Jet	Direct CAP application increase the cytotoxic effects of 5-Fluorouracil [73].

## Data Availability

Not applicable.

## Abbreviations

OH: Hydroxyl
5-FU: 5-fluorouracil
CAP: Cold atmospheric plasma
CHF: Congestive heart failure
CIS: Cisplatin
CNS: Central nervous system
CSC: Cancer stem cells
DBDs: Dielectric barrier discharge devices
DOX: Doxorubicin
DPD: Dihydropyrimidine dehydrogenase
EPI: Epirubicin
ER: Estrogen receptor
GBM: Glioblastoma multiforme
GSH: Gluthatione
H_2_O_2_: Hydrogen peroxide
HCC: Hepatocellular carcinoma
LV: Left ventricle
MGMT: Methylguanine-DNA methyl transferase
N: Nitrogen
NO: Nitric oxide
O: Oxygen
O_2_: Singlet-delta oxygen
O_2_^−^: Superoxide
OHOO: Hydroxyl peroxide
ONOO^−^: peroxynitrite
PCL: Plasma-conditioned liquid
PTX: Paclitaxel
RNS: Reactive nitrogen species
RONS: Reactive oxygen and nitrogen species
TMZ: Temozolomide
TOP2A: Topoisomerase II
TS: Thymidylate synthase

## References

[1] Ward R.A., Fawell S., Floc’h N., Flemington V., McKerrecher D., Smith P.D. (2021). Challenges and Opportunities in Cancer Drug Resistance. Chem. Rev..

[2] Vasan N., Baselga J., Hyman D.M. (2019). A View on Drug Resistance in Cancer. Nature.

[3] Braný D., Dvorská D., Halašová E., Škovierová H. (2020). Cold Atmospheric Plasma: A Powerful Tool for Modern Medicine. Int. J. Mol. Sci..

[4] Labrie M., Brugge J.S., Mills G.B., Zervantonakis I.K. (2022). Therapy Resistance: Opportunities Created by Adaptive Responses to Targeted Therapies in Cancer. Nat. Rev. Cancer.

[5] Chitcholtan K., Asselin E., Parent S., Sykes P.H., Evans J.J. (2013). Differences in Growth Properties of Endometrial Cancer in Three Dimensional (3d) Culture and 2d Cell Monolayer. Exp. Cell Res..

[6] Van der Worp H.B., Howells D.W., Sena E.S., Porritt M.J., Rewell S., O’Collins V., Macleod M.R. (2010). Can Animal Models of Disease Reliably Inform Human Studies?. PLoS Med..

[7] Kimlin L.C., Casagrande G., Virador V.M. (2013). In Vitro Three-Dimensional (3d) Models in Cancer Research: An Update. Mol. Carcinog..

[8] De Luca A., Raimondi L., Salamanna F., Carina V., Costa V., Bellavia D., Alessandro R., Fini M., Giavaresi G. (2018). Relevance of 3d Culture Systems to Study Osteosarcoma Environment. J. Exp. Clin. Cancer Res..

[9] Tornin J., Labay C., Tampieri F., Ginebra M.-P., Canal C. (2021). Evaluation of the Effects of Cold Atmospheric Plasma and Plasma-Treated Liquids in Cancer Cell Cultures. Nat. Protoc..

[10] Bernhardt T., Semmler M.L., Schäfer M., Bekeschus S., Emmert S., Boeckmann L. (2019). Plasma Medicine: Applications of Cold Atmospheric Pressure Plasma in Dermatology. Oxid. Med. Cell Longev.

[11] Dubuc A., Monsarrat P., Virard F., Merbahi N., Sarrette J.-P., Laurencin-Dalicieux S., Cousty S. (2018). Use of Cold-Atmospheric Plasma in Oncology: A Concise Systematic Review. Adv. Med. Oncol..

[12] Izadjoo M., Zack S., Kim H., Skiba J. (2018). Medical Applications of Cold Atmospheric Plasma: State of the Science. J. Wound Care.

[13] Attri P., Yusupov M., Park J.H., Lingamdinne L.P., Koduru J.R., Shiratani M., Choi E.H., Bogaerts A. (2016). Mechanism and Comparison of Needle-Type Non-Thermal Direct and Indirect Atmospheric Pressure Plasma Jets on the Degradation of Dyes. Sci. Rep..

[14] Lu X., Naidis G.V., Laroussi M., Reuter S., Graves D.B., Ostrikov K. (2016). Reactive Species in Non-Equilibrium Atmospheric-Pressure Plasmas: Generation, Transport, and Biological Effects. Phys. Rep..

[15] Rakesh Ruchel K., Bailung H., Shahzad A. (2021). Cold Atmospheric Pressure Plasma Technology for Biomedical Application. Plasma Science and Technology.

[16] Khlyustova A., Labay C., Machala Z., Ginebra M.-P., Canal C. (2019). Important Parameters in Plasma Jets for the Production of Rons in Liquids for Plasma Medicine: A Brief Review. Front. Chem. Sci. Eng..

[17] Lis K.A., Kehrenberg C., Boulaaba A., von Köckritz-Blickwede M., Binder S., Li Y., Zimmermann J.L., Pfeifer Y., Ahlfeld B. (2018). Inactivation of Multidrug-Resistant Pathogens and Y. Enterocolitica with Cold Atmospheric Pressure Plasma on Stainless Steel Surfaces. Int. J. Antimicrob. Agents.

[18] Ercan U.K., Ibiş F., Dikyol C., Horzum N., Karaman O., Yıldırım Ç., Çukur E., Demirci E.A. (2018). Prevention of Bacterial Colonization on Non-Thermal Atmospheric Plasma Treated Surgical Sutures for Control and Prevention of Surgical Site Infections. PLoS ONE.

[19] Guo L., Xu R., Gou L., Liu Z., Zhao Y., Liu D., Zhang L., Chen H., Kong M.G. (2018). Mechanism of Virus Inactivation by Cold Atmospheric-Pressure Plasma and Plasma-Activated Water. Appl. Env. Microbiol..

[20] Chen Z., Garcia G., Arumugaswami V., Wirz R.E. (2020). Cold Atmospheric Plasma for SARS-CoV-2 Inactivation. Phys. Fluids.

[21] Borges A.C., de Morais Gouvêa Lima G., Nishime T.M.C., Gontijo A.V.L., Kostov K.G., Koga-Ito C.Y. (2018). Amplitude-Modulated Cold Atmospheric Pressure Plasma Jet for Treatment of Oral Candidiasis: In Vivo Study. PLoS ONE.

[22] Tan F., Rui X., Xiang X., Yu Z., Al-Rubeai M. (2021). Multimodal Treatment Combining Cold Atmospheric Plasma and Acidic Fibroblast Growth Factor for Multi-Tissue Regeneration. FASEB J..

[23] Amini M.R., Hosseini M.S., Fatollah S., Mirpour S., Ghoranneviss M., Larijani B., Mohajeri-Tehrani M.R., Khorramizadeh M.R. (2020). Beneficial Effects of Cold Atmospheric Plasma on Inflammatory Phase of Diabetic Foot Ulcers; a Randomized Clinical Trial. J. Diabetes Metab. Disord..

[24] Duchesne C., Banzet S., Lataillade J., Rousseau A., Frescaline N. (2019). Cold Atmospheric Plasma Modulates Endothelial Nitric Oxide Synthase Signalling and Enhances Burn Wound Neovascularisation. J. Pathol..

[25] Borchardt T., Ernst J., Helmke A., Tanyeli M., Schilling A.F., Felmerer G., Viöl W. (2017). Effect of Direct Cold Atmospheric Plasma (Dicap) on Microcirculation of Intact Skin in a Controlled Mechanical Environment. Microcirculation.

[26] Nomura Y., Takamatsu T., Kawano H., Miyahara H., Okino A., Yoshida M., Azuma T. (2017). Investigation of Blood Coagulation Effect of Nonthermal Multigas Plasma Jet in Vitro and in Vivo. J. Surg. Res..

[27] Haertel B., von Woedtke T., Weltmann K.D., Lindequist U. (2014). Non-Thermal Atmospheric-Pressure Plasma Possible Application in Wound Healing. Biomol. Ther..

[28] Lee J.-H., Jeong W.-S., Seo S.-J., Kim H.-W., Kim K.-N., Choi E.-H., Kim K.-M. (2017). Non-Thermal Atmospheric Pressure Plasma Functionalized Dental Implant for Enhancement of Bacterial Resistance and Osseointegration. Dent. Mater..

[29] Duske K., Jablonowski L., Koban I., Matthes R., Holtfreter B., Sckell A., Nebe J.B., von Woedtke T., Weltmann K.D., Kocher T. (2015). Cold Atmospheric Plasma in Combination with Mechanical Treatment Improves Osteoblast Growth on Biofilm Covered Titanium Discs. Biomaterials.

[30] Omori M., Tsuchiya S., Hara K., Kuroda K., Hibi H., Okido M., Ueda M. (2015). A New Application of Cell-Free Bone Regeneration: Immobilizing Stem Cells from Human Exfoliated Deciduous Teeth-Conditioned Medium onto Titanium Implants Using Atmospheric Pressure Plasma Treatment. Stem. Cell Res. Ther..

[31] Won H.-R., Kang S.U., Kim H.J., Jang J.Y., Shin Y.S., Kim C.-H. (2018). Non-Thermal Plasma Treated Solution with Potential as a Novel Therapeutic Agent for Nasal Mucosa Regeneration. Sci. Rep..

[32] Eisenhauer P., Chernets N., Song Y., Dobrynin D., Pleshko N., Steinbeck M.J., Freeman T.A. (2016). Chemical Modification of Extracellular Matrix by Cold Atmospheric Plasma-Generated Reactive Species Affects Chondrogenesis and Bone Formation. J. Tissue Eng. Regen. Med..

[33] Xiong Z., Zhao S., Yan X. (2019). Nerve Stem Cell Differentiation by a One-Step Cold Atmospheric Plasma Treatment in Vitro. J. Vis. Exp..

[34] Tan F., Fang Y., Zhu L., Al-Rubeai M. (2020). Controlling Stem Cell Fate Using Cold Atmospheric Plasma. Stem. Cell Res. Ther..

[35] Canady Helios Cold Plasma Scalpel Treatment at the Surgical Margin and Macroscopic Tumor Sites. https://ClinicalTrials.gov/show/NCT04267575.

[36] Mateu-Sanz M., Tornin J., Ginebra M.P., Canal C. (2021). Cold Atmospheric Plasma: A New Strategy Based Primarily on Oxidative Stress for Osteosarcoma Therapy. J. Clin. Med..

[37] Bauer G., Sersenová D., Graves D.B., Machala Z. (2019). Cold Atmospheric Plasma and Plasma-Activated Medium Trigger Rons-Based Tumor Cell Apoptosis. Sci. Rep..

[38] Tornin J., Mateu-Sanz M., Rodríguez A., Labay C., Rodríguez R., Canal C. (2019). Pyruvate Plays a Main Role in the Antitumoral Selectivity of Cold Atmospheric Plasma in Osteosarcoma. Sci. Rep..

[39] Mateu-Sanz M., Ginebra M.-P., Tornín J., Canal C. (2022). Cold Atmospheric Plasma Enhances Doxorubicin Selectivity in Metastasic Bone Cancer. Free. Radic. Biol. Med..

[40] Mateu-Sanz M., Tornín J., Brulin B., Khlyustova A., Ginebra M.-P., Layrolle P., Canal C. (2020). Cold Plasma-Treated Ringer’s Saline: A Weapon to Target Osteosarcoma. Cancers.

[41] Freund E., Liedtke K.R., van der Linde J., Metelmann H.R., Heidecke C.D., Partecke L.I., Bekeschus S. (2019). Physical Plasma-Treated Saline Promotes an Immunogenic Phenotype in Ct26 Colon Cancer Cells in Vitro and in Vivo. Sci. Rep..

[42] Bauer G. (2019). The Synergistic Effect between Hydrogen Peroxide and Nitrite, Two Long-Lived Molecular Species from Cold Atmospheric Plasma, Triggers Tumor Cells to Induce Their Own Cell Death. Redox Biol..

[43] Lukes P., Dolezalova E., Sisrova I., Clupek M. (2014). Aqueous-Phase Chemistry and Bactericidal Effects from an Air Discharge Plasma in Contact with Water: Evidence for the Formation of Peroxynitrite through a Pseudo-Second-Order Post-Discharge Reaction of H2o 2 and Hno2. Plasma Sources Sci. Technol..

[44] Solé-Martí X., Espona-Noguera A., Ginebra M.-P., Canal C. (2021). Plasma-Conditioned Liquids as Anticancer Therapies in Vivo: Current State and Future Directions. Cancers.

[45] Girard P.-M., Arbabian A., Fleury M., Bauville G., Puech V., Dutreix M., Sousa J.S. (2016). Synergistic Effect of H_2_O_2_ and No_2_ in Cell Death Induced by Cold Atmospheric He Plasma. Sci. Rep..

[46] Bauer G. (2019). Cold Atmospheric Plasma and Plasma-Activated Medium: Antitumor Cell Effects with Inherent Synergistic Potential. Plasma Med..

[47] Bekeschus S., Liebelt G., Menz J., Berner J., Sagwal S.K., Wende K., Weltmann K.-D., Boeckmann L., von Woedtke T., Metelmann H.-R. (2021). Tumor Cell Metabolism Correlates with Resistance to Gas Plasma Treatment: The Evaluation of Three Dogmas. Free. Radic. Biol. Med..

[48] de Sa Junior P.L., Câmara D.A.D., Porcacchia A.S., Fonseca P.M.M., Jorge S.D., Araldi R.P., Ferreira A.K. (2017). The Roles of Ros in Cancer Heterogeneity and Therapy. Oxid. Med. Cell. Longev..

[49] Kurake N., Tanaka H., Ishikawa K., Kondo T., Sekine M., Nakamura K., Kajiyama H., Kikkawa F., Mizuno M., Hori M. (2016). Cell Survival of Glioblastoma Grown in Medium Containing Hydrogen Peroxide and/or Nitrite, or in Plasma-Activated Medium. Arch. Biochem. Biophys..

[50] Mahdikia H., Shokri B., Majidzadeh K.A. (2021). The Feasibility Study of Plasma-Activated Water as a Physical Therapy to Induce Apoptosis in Melanoma Cancer Cells in-Vitro. Iran. J. Pharm. Res..

[51] Raud S., Raud J., Jõgi I., Piller C.-T., Plank T., Talviste R., Teesalu T., Vasar E. (2021). The Production of Plasma Activated Water in Controlled Ambient Gases and Its Impact on Cancer Cell Viability. Plasma Chem. Plasma Process..

[52] Tornín J., Villasante A., Solé-Martí X., Ginebra M.-P., Canal C. (2021). Osteosarcoma Tissue-Engineered Model Challenges Oxidative Stress Therapy Revealing Promoted Cancer Stem Cell Properties. Free. Radic. Biol. Med..

[53] Sato Y., Yamada S., Takeda S., Hattori N., Nakamura K., Tanaka H., Mizuno M., Hori M., Kodera Y. (2018). Effect of Plasma-Activated Lactated Ringer’s Solution on Pancreatic Cancer Cells in Vitro and in Vivo. Ann. Surg. Oncol..

[54] Privat-Maldonado A., Gorbanev Y., Dewilde S., Smits E., Bogaerts A. (2018). Reduction of Human Glioblastoma Spheroids Using Cold Atmospheric Plasma: The Combined Effect of Short- and Long-Lived Reactive Species. Cancers.

[55] Zhou X., Cai D., Xiao S., Ning M., Zhou R., Zhang S., Chen X., Ostrikov K., Dai X. (2020). Invivopen: A Novel Plasma Source for in Vivo Cancer Treatment. J. Cancer.

[56] Motaln H., Recek N., Rogelj B. (2021). Intracellular Responses Triggered by Cold Atmospheric Plasma and Plasma-Activated Media in Cancer Cells. Molecules.

[57] Kumari S., Badana A.K., Murali M.G., Shailender G., Malla R. (2018). Reactive Oxygen Species: A Key Constituent in Cancer Survival. Biomark Insights.

[58] Köritzer J., Boxhammer V., Schäfer A., Shimizu T., Klämpfl T.G., Li Y.-F., Welz C., Schwenk-Zieger S., Morfill G.E., Zimmermann J.L. (2013). Restoration of Sensitivity in Chemo-Resistant Glioma Cells by Cold Atmospheric Plasma. PLoS ONE.

[59] Gjika E., Pal-Ghosh S., Kirschner M.E., Lin L., Sherman J.H., Stepp M.A., Keidar M. (2020). Combination Therapy of Cold Atmospheric Plasma (Cap) with Temozolomide in the Treatment of U87mg Glioblastoma Cells. Sci. Rep..

[60] Soni V., Adhikari M., Simonyan H., Lin L., Sherman J.H., Young C.N., Keidar M. (2021). In Vitro and in Vivo Enhancement of Temozolomide Effect in Human Glioblastoma by Non-Invasive Application of Cold Atmospheric Plasma. Cancers.

[61] Shaw P., Kumar N., Privat-Maldonado A., Smits E., Bogaerts A. (2021). Cold Atmospheric Plasma Increases Temozolomide Sensitivity of Three-Dimensional Glioblastoma Spheroids Via Oxidative Stress-Mediated DNA Damage. Cancers.

[62] Sagwal S.K., Pasqual-Melo G., Bodnar Y., Gandhirajan R.K., Bekeschus S. (2018). Combination of Chemotherapy and Physical Plasma Elicits Melanoma Cell Death Via Upregulation of Slc22a16. Cell Death Dis..

[63] Pefani-Antimisiari K., Athanasopoulos D.K., Marazioti A., Sklias K., Rodi M., de Lastic A.-L., Mouzaki A., Svarnas P., Antimisiaris S.G. (2021). Synergistic Effect of Cold Atmospheric Pressure Plasma and Free or Liposomal Doxorubicin on Melanoma Cells. Sci. Rep..

[64] Zhang H., Xu S., Zhang J., Li B., Liu D., Guo L., Liu Z., Xu D. (2021). Synergistic Anticancer Effects of Different Combinations of He+O2 Plasma Jet and Doxorubicin on A375 Melanoma Cells. Plasma Process. Polym..

[65] Zahedian S., Hekmat A., Tackallou S.H., Ghoranneviss M. (2022). The Impacts of Prepared Plasma-Activated Medium (Pam) Combined with Doxorubicin on the Viability of Mcf-7 Breast Cancer Cells: A New Cancer Treatment Strategy. Rep. Biochem. Mol. Biol..

[66] Brunner T.F., Probst F.A., Troeltzsch M., Schwenk-Zieger S., Zimmermann J.L., Morfill G., Becker S., Harréus U., Welz C. (2022). Primary Cold Atmospheric Plasma Combined with Low Dose Cisplatin as a Possible Adjuvant Combination Therapy for Hnscc Cells-an in-Vitro Study. Head Face Med..

[67] Lee C.-M., Jeong Y.-I., Kook M.-S., Kim B.-H. (2020). Combinatorial Effect of Cold Atmosphere Plasma (Cap) and the Anticancer Drug Cisplatin on Oral Squamous Cell Cancer Therapy. Int. J. Mol. Sci..

[68] Li Y., Tang T., Lee H., Song K. (2021). Selective Anti-Cancer Effects of Plasma-Activated Medium and Its High Efficacy with Cisplatin on Hepatocellular Carcinoma with Cancer Stem Cell Characteristics. Int. J. Mol. Sci..

[69] Mihai C.-T., Mihaila I., Pasare M.A., Pintilie R.M., Ciorpac M., Topala I. (2022). Cold Atmospheric Plasma-Activated Media Improve Paclitaxel Efficacy on Breast Cancer Cells in a Combined Treatment Model. Curr. Issues Mol. Biol..

[70] Park S., Kim H., Ji H.W., Kim H.W., Yun S.H., Choi E.H., Kim S.J. (2019). Cold Atmospheric Plasma Restores Paclitaxel Sensitivity to Paclitaxel-Resistant Breast Cancer Cells by Reversing Expression of Resistance-Related Genes. Cancers.

[71] Lee S., Lee H., Jeong D., Ham J., Park S., Choi E.H., Kim S.J. (2017). Cold atmospheric plasma restores tamoxifen sensitivity in resistant MCF-7 breast cancer cell. Free. Radic. Biol. Med..

[72] Yang H., Lu R., Xian Y., Gan L., Lu X., Yang X. (2015). Effects of Atmospheric Pressure Cold Plasma on Human Hepatocarcinoma Cell and Its 5-Fluorouracil Resistant Cell Line. Phys. Plasmas.

[73] Jones O., Cheng X., Murthy S.R.K., Ly L., Zhuang T., Basadonna G., Keidar M., Canady J. (2021). The synergistic effect of Canady Helios cold atmospheric plasma and a FOLFIRINOX regimen for the treatment of cholangiocarcinoma in vitro. Sci. Rep..

[74] Moody C.L., Wheelhouse R.T. (2014). The Medicinal Chemistry of Imidazotetrazine Prodrugs. Pharmaceuticals.

[75] Lee S.Y. (2016). Temozolomide Resistance in Glioblastoma Multiforme. Genes Dis..

[76] De Vleeschouwer S. (2017). Glioblastoma.

[77] Hanif F., Muzaffar K., Perveen K., Malhi S.M., Simjee S.U. (2017). Glioblastoma Multiforme: A Review of Its Epidemiology and Pathogenesis through Clinical Presentation and Treatment. Asian Pac. J. Cancer Prev. APJCP.

[78] Haar C.P., Hebbar P., Wallace G.C., Das A., Vandergrift W.A., Smith J.A., Giglio P., Patel S.J., Ray S.K., Banik N.L. (2012). Drug Resistance in Glioblastoma: A Mini Review. Neurochem. Res..

[79] Danson S.J., Middleton M.R. (2001). Temozolomide: A Novel Oral Alkylating Agent. Expert Rev. Anticancer..

[80] Zhang J., Stevens M.F., Laughton C.A., Madhusudan S., Bradshaw T.D. (2010). Acquired Resistance to Temozolomide in Glioma Cell Lines: Molecular Mechanisms and Potential Translational Applications. Oncology.

[81] Alonso M.M., Gomez-Manzano C., Bekele B.N., Yung W.K., Fueyo J. (2007). Adenovirus-Based Strategies Overcome Temozolomide Resistance by Silencing the O6-Methylguanine-DNA Methyltransferase Promoter. Cancer Res..

[82] Hermisson M., Klumpp A., Wick W., Wischhusen J., Nagel G., Roos W., Kaina B., Weller M. (2006). O6-Methylguanine DNA Methyltransferase and P53 Status Predict Temozolomide Sensitivity in Human Malignant Glioma Cells. J. Neurochem..

[83] Kohsaka S., Wang L., Yachi K., Mahabir R., Narita T., Itoh T., Tanino M., Kimura T., Nishihara H., Tanaka S. (2012). Stat3 Inhibition Overcomes Temozolomide Resistance in Glioblastoma by Downregulating Mgmt Expression. Mol. Cancer.

[84] Liu G., Yuan X., Zeng Z., Tunici P., Ng H., Abdulkadir I.R., Lu L., Irvin D., Black K.L., Yu J.S. (2006). Analysis of Gene Expression and Chemoresistance of Cd133+ Cancer Stem Cells in Glioblastoma. Mol. Cancer.

[85] Sarkar C., Suri V., Jha P., Sharma M.C. (2011). O6 -Methylguanine DNA Methyltransferase Gene Promoter Methylation in High-Grade Gliomas: A Review of Current Status. Neurol. India.

[86] Zhang W.-B., Wang Z., Shu F., Jin Y.-H., Liu H.-Y., Wang Q.-J., Yang Y. (2010). Activation of Amp-Activated Protein Kinase by Temozolomide Contributes to Apoptosis in Glioblastoma Cells Via P53 Activation and Mtorc1 Inhibition. J. Biol. Chem..

[87] Rocha C.R.R., Kajitani G.S., Quinet A., Fortunato R.S., Menck C.F.M. (2016). Nrf2 and Glutathione Are Key Resistance Mediators to Temozolomide in Glioma and Melanoma Cells. Oncotarget.

[88] Zhu Z., Du S., Du Y., Ren J., Ying G., Yan Z. (2017). Glutathione Reductase Mediates Drug Resistance in Glioblastoma Cells by Regulating Redox Homeostasis. J. Neurochem..

[89] Hulst M.B., Grocholski T., Neefjes J.J.C., van Wezel G.P., Metsä-Ketelä M. (2021). Anthracyclines: Biosynthesis, engineering and clinical applications. Nat. Prod. Rep..

[90] Sritharan S., Sivalingam N. (2021). A Comprehensive Review on Time-Tested Anticancer Drug Doxorubicin. Life Sci..

[91] Al-Malky H.S., Al Harthi S.E., Osman A.M. (2020). Major Obstacles to Doxorubicin Therapy: Cardiotoxicity and Drug Resistance. J. Oncol. Pharm. Pr..

[92] Ling G., Wang X., Tan N., Cao J., Li W., Zhang Y., Jiang J., Sun Q., Jiang Y., Wang W. (2022). Mechanisms and Drug Intervention for Doxorubicin-Induced Cardiotoxicity Based on Mitochondrial Bioenergetics. Oxidative Med. Cell. Longev..

[93] Watson C., Gadikota H., Barlev A., Beckerman R. (2022). A review of the risks of long-term consequences associated with components of the CHOP chemotherapy regimen. J. Drug Assess..

[94] Mirzaei S., Gholami M.H., Hashemi F., Zabolian A., Farahani M.V., Hushmandi K., Zarrabi A., Goldman A., Ashrafizadeh M., Orive G. (2022). Advances in Understanding the Role of P-Gp in Doxorubicin Resistance: Molecular Pathways, Therapeutic Strategies, and Prospects. Drug Discov Today.

[95] Genovese I., Ilari A., Assaraf Y.G., Fazi F., Colotti G. (2017). Not Only P-Glycoprotein: Amplification of the Abcb1-Containing Chromosome Region 7q21 Confers Multidrug Resistance Upon Cancer Cells by Coordinated Overexpression of an Assortment of Resistance-Related Proteins. Drug Resist Updat..

[96] Jayaraj R., Nayagam S.g., Kar A., Sathyakumar S., Mohammed H., Smiti M., Sabarimurugan S., Kumarasamy C., Priyadharshini T., Gothandam K.M. (2019). Clinical Theragnostic Relationship between Drug-Resistance Specific Mirna Expressions, Chemotherapeutic Resistance, and Sensitivity in Breast Cancer: A Systematic Review and Meta-Analysis. Cells.

[97] Si Z., Zhong Y., Lao S., Wu Y., Zhong G., Zeng W. (2022). The Role of Mirnas in the Resistance of Anthracyclines in Breast Cancer: A Systematic Review. Front Oncol..

[98] Menéndez S., Gallego B., Murillo D., Rodríguez A., Rodríguez R. (2021). Cancer Stem Cells as a Source of Drug Resistance in Bone Sarcomas. J. Clin. Med..

[99] Martins-Neves S.R., Sampaio-Ribeiro G., Gomes C.M.F. (2022). Chemoresistance-Related Stem Cell Signaling in Osteosarcoma and Its Plausible Contribution to Poor Therapeutic Response: A Discussion That Still Matters. Int. J. Mol. Sci..

[100] Singh M.S., Tammam S.N., Boushehri M.A.S., Lamprecht A. (2017). MDR in cancer: Addressing the underlying cellular alterations with the use of nanocarriers. Pharmacol. Res..

[101] Tzelepis K., Koike-Yusa H., De Braekeleer E., Li Y., Metzakopian E., Dovey O.M., Mupo A., Grinkevich V., Li M., Mazan M. (2016). A Crispr Dropout Screen Identifies Genetic Vulnerabilities and Therapeutic Targets in Acute Myeloid Leukemia. Cell Rep..

[102] Yang F., Teves S.S., Kemp C.J., Henikoff S. (2014). Doxorubicin, DNA Torsion, and Chromatin Dynamics. Biochim Biophys Acta.

[103] Tarpgaard L.S., Qvortrup C., Nielsen S.L., Stenvang J., Detlefsen S., Brünner N., Pfeiffer P. (2021). New use for old drugs: Epirubicin in colorectal cancer. Acta Oncol..

[104] Zhang J., Jiang H., Bao G., Zhang G., Wang H., Wang X. (2021). Effectiveness and safety of pegylated liposomal doxorubicin versus epirubicin as neoadjuvant or adjuvant chemotherapy for breast cancer: A real-world study. BMC Cancer.

[105] Steele N.G., Chakrabarti J., Wang J., Biesiada J., Holokai L., Chang J., Nowacki L.M., Hawkins J., Mahe M., Sundaram N. (2019). An Organoid-Based Preclinical Model of Human Gastric Cancer. Cell. Mol. Gastroenterol. Hepatol..

[106] Liu J.-J., Tang W., Fu M., Gong X.-Q., Kong L., Yao X.-M., Jing M., Cai F.-Y., Li X.-T., Ju R.-J. (2019). Development of R8 modified epirubicin–dihydroartemisinin liposomes for treatment of non-small-cell lung cancer. Artif. Cells Nanomedicine Biotechnol..

[107] Armenian S., Bhatia S. (2018). Predicting and Preventing Anthracycline-Related Cardiotoxicity. Am. Soc. Clin. Oncol. Educ. Book.

[108] Armstrong G.T., Oeffinger K.C., Chen Y., Kawashima T., Yasui Y., Leisenring W., Stovall M., Chow E., Sklar C.A., Mulrooney D.A. (2013). Modifiable Risk Factors and Major Cardiac Events Among Adult Survivors of Childhood Cancer. J. Clin. Oncol..

[109] (2005). Effects of Chemotherapy and Hormonal Therapy for Early Breast Cancer on Recurrence and 15-Year Survival: An Overview of the Randomised Trials. Lancet.

[110] Scully R.E., Lipshultz S.E. (2007). Anthracycline cardiotoxicity in long-term survivors of childhood cancer. Cardiovasc. Toxicol..

[111] Calvanese V., Lara E., Suárez-Álvarez B., Abu Dawud R., Vázquez-Chantada M., Martínez-Chantar M.L., Embade N., López-Nieva P., Horrillo A., Hmadcha A. (2010). Sirtuin 1 regulation of developmental genes during differentiation of stem cells. Proc. Natl. Acad. Sci. USA.

[112] Robinson E.L., Azodi M., Heymans S., Heggermont W. (2020). Anthracycline-Related Heart Failure: Certain Knowledge and Open Questions: Where Do We Stand with Chemotherapyinduced Cardiotoxicity?. Curr. Heart Fail Rep..

[113] Vitale D., Caon I., Parnigoni A., Sevic I., Spinelli F., Icardi A., Passi A., Vigetti D., Alaniz L. (2021). Initial Identification of UDP-Glucose Dehydrogenase as a Prognostic Marker in Breast Cancer Patients, Which Facilitates Epirubicin Resistance and Regulates Hyaluronan Synthesis in MDA-MB-231 Cells. Biomolecules.

[114] Li Z., Li C., Wu Q., Tu Y., Wang C., Yu X., Li B., Wang Z., Sun S., Sun S. (2021). MEDAG enhances breast cancer progression and reduces epirubicin sensitivity through the AKT/AMPK/mTOR pathway. Cell Death Dis..

[115] Qiong L., Yin J. (2021). Orosomucoid 1 promotes epirubicin resistance in breast cancer by upregulating the expression of matrix metalloproteinases 2 and 9. Bioengineered.

[116] Kopacz-Bednarska A., Król T. (2022). Cisplatin—Properties and Clinical Application. Oncol. Clin. Pract..

[117] Ghosh S. (2019). Cisplatin: The First Metal Based Anticancer Drug. Bioorg. Chem..

[118] Crona D.J., Faso A., Nishijima T.F., McGraw K.A., Galsky M.D., Milowsky M.I. (2017). A Systematic Review of Strategies to Prevent Cisplatin-Induced Nephrotoxicity. Oncologist.

[119] Duan Z., Cai G., Li J., Chen X. (2020). Cisplatin-Induced Renal Toxicity in Elderly People. Adv. Med. Oncol..

[120] Manohar S., Leung N. (2018). Cisplatin Nephrotoxicity: A Review of the Literature. J. Nephrol..

[121] Lugones Y., Loren P., Salazar L.A. (2022). Cisplatin Resistance: Genetic and Epigenetic Factors Involved. Biomolecules.

[122] Wang L., Zhao X., Fu J., Xu W., Yuan J. (2021). The Role of Tumour Metabolism in Cisplatin Resistance. Front. Mol. Biosci..

[123] Han Y., Wen P., Li J., Kataoka K. (2022). Targeted nanomedicine in cisplatin-based cancer therapeutics. J. Control. Release.

[124] Sun C.-Y., Zhang Q.-Y., Zheng G.-J., Feng B. (2019). Phytochemicals: Current strategy to sensitize cancer cells to cisplatin. Biomed. Pharmacother..

[125] Sun C.-Y., Nie J., Huang J.-P., Zheng G.-J., Feng B. (2019). Targeting STAT3 inhibition to reverse cisplatin resistance. Biomed. Pharmacother..

[126] Sharifi-Rad J., Quispe C., Patra J., Singh Y., Panda M., Das G., Adetunji C., Michael O., Sytar O., Polito L. (2021). Paclitaxel: Application in Modern Oncology and Nanomedicine-Based Cancer Therapy. Oxid. Med. Cell Longev..

[127] Zhu L., Chen L. (2019). Progress in Research on Paclitaxel and Tumor Immunotherapy. Cell Mol. Biol. Lett..

[128] Weaver B.A. (2014). How Taxol/Paclitaxel Kills Cancer Cells. Mol. Biol. Cell.

[129] Woods C.M., Zhu J., McQueney P.A., Bollag D., Lazarides E. (1995). Taxol-Induced Mitotic Block Triggers Rapid Onset of a P53-Independent Apoptotic Pathway. Mol. Med..

[130] Shao Z., Zhao H. (2014). Manipulating Natural Product Biosynthetic Pathways Via DNA Assembler. Curr. Protoc. Chem. Biol..

[131] Murray S., Briasoulis E., Linardou H., Bafaloukos D., Papadimitriou C. (2012). Taxane resistance in breast cancer: Mechanisms, predictive biomarkers and circumvention strategies. Cancer Treat. Rev..

[132] (2015). Septin Cooperation with Tubulin Polyglutamylation Contributes to Cancer Cell Adaptation to Taxanes. Oncotarget.

[133] Liu Z., Zhu G., Getzenberg R.H., Veltri R.W. (2015). The Upregulation of PI3K/Akt and MAP Kinase Pathways is Associated with Resistance of Microtubule-Targeting Drugs in Prostate Cancer. J. Cell. Biochem..

[134] Dong C., Wu J., Chen Y., Nie J., Chen C. (2021). Activation of PI3K/AKT/mTOR Pathway Causes Drug Resistance in Breast Cancer. Front. Pharmacol..

[135] Quirke V.M. (2017). Tamoxifen from Failed Contraceptive Pill to Best-Selling Breast Cancer Medicine: A Case-Study in Pharmaceutical Innovation. Front Pharm..

[136] Chang M. (2012). Tamoxifen Resistance in Breast Cancer. Biomoleculs.

[137] Longley D.B., Harkin D.P., Johnston P.G. (2003). 5-Fluorouracil: Mechanisms of action and clinical strategies. Nat. Rev. Cancer.

[138] Azwar S., Seow H.F., Abdullah M., Jabar M.F., Mohtarrudin N. (2021). Recent Updates on Mechanisms of Resistance to 5-Fluorouracil and Reversal Strategies in Colon Cancer Treatment. Biology.

[139] Ghafouri-Fard S., Abak A., Tondro Anamag F., Shoorei H., Fattahi F., Javadinia S.A., Taheri M. (2021). 5-Fluorouracil: A Narrative Review on the Role of Regulatory Mechanisms in Driving Resistance to This Chemotherapeutic Agent. Front. Oncol..

[140] Blondy S., David V., Verdier M., Mathonnet M., Perraud A., Christou N. (2020). 5-Fluorouracil resistance mechanisms in colorectal cancer: From classical pathways to promising processes. Cancer Sci..

[141] Yan D., Cui H., Zhu W., Nourmohammadi N., Milberg J., Zhang L.G., Sherman J.H., Keidar M. (2017). The Specific Vulnerabilities of Cancer Cells to the Cold Atmospheric Plasma-Stimulated Solutions. Sci. Rep..

[142] Yan D., Talbot A., Nourmohammadi N., Sherman J.H., Cheng X., Keidar M. (2015). Toward Understanding the Selective Anticancer Capacity of Cold Atmospheric Plasma—A Model Based on Aquaporins. Biointerphases.

[143] Van Der Paal J., Verheyen C., Neyts E.C., Bogaerts A. (2017). Hampering Effect of Cholesterol on the Permeation of Reactive Oxygen Species through Phospholipids Bilayer: Possible Explanation for Plasma Cancer Selectivity. Sci. Rep..

[144] Bauer G., Graves D.B. (2016). Mechanisms of Selective Antitumor Action of Cold Atmospheric Plasma-Derived Reactive Oxygen and Nitrogen Species. Plasma Process. Polym..

[145] Bauer G. (2018). Signal Amplification by Tumor Cells: Clue to the Understanding of the Antitumor Effects of Cold Atmospheric Plasma and Plasma-Activated Medium. IEEE Trans. Radiat. Plasma Med. Sci..

[146] Bauer G. (2018). Targeting Protective Catalase of Tumor Cells with Cold Atmospheric Plasma—Activated Medium (Pam). Anti-Cancer Agents Med. Chem..

[147] Trachootham D., Alexandre J., Huang P. (2009). Targeting Cancer Cells by Ros-Mediated Mechanisms: A Radical Therapeutic Approach?. Nat. Rev. Drug Discov..

[148] Ghanbari Movahed Z., Rastegari-Pouyani M., Mohammadi M.H., Mansouri K. (2019). Cancer Cells Change Their Glucose Metabolism to Overcome Increased Ros: One Step from Cancer Cell to Cancer Stem Cell?. Biomed. Pharmacother..

[149] Jablonowski L., Kocher T., Schindler A., Müller K., Dombrowski F., Von Woedtke T., Arnold T., Lehmann A., Rupf S., Evert M. (2019). Side Effects by Oral Application of Atmospheric Pressure Plasma on the Mucosa in Mice. PLoS ONE.

[150] Baik K.Y., Huh Y.H., Kim Y.H., Kim J., Kim M.S., Park H.-K., Choi E.H., Park B. (2017). The Role of Free Radicals in Hemolytic Toxicity Induced by Atmospheric-Pressure Plasma Jet. Oxidative Med. Cell. Longev..

[151] Visvader J.E., Lindeman G.J. (2012). Cancer Stem Cells: Current Status and Evolving Complexities. Cell Stem Cell.

[152] Visvader J.E. (2011). Cells of Origin in Cancer. Nature.

[153] Shijie D., Li C., Cheng N., Cui X., Xu X., Zhou G. (2015). Redox Regulation in Cancer Stem Cells. Oxidative Med. Cell. Longev..

[154] Dayem A.A., Choi H.-Y., Kim J.-H., Cho S.-G. (2010). Role of Oxidative Stress in Stem, Cancer, and Cancer Stem Cells. Cancers.

[155] Okon I.S., Zou M.-H. (2015). Mitochondrial ROS and cancer drug resistance: Implications for therapy. Pharmacol. Res..

[156] Kahroba H., Shirmohamadi M., Hejazi M.S., Samadi N. (2019). The Role of Nrf2 signaling in cancer stem cells: From stemness and self-renewal to tumorigenesis and chemoresistance. Life Sci..

[157] Shi X., Zhang Y., Zheng J., Pan J. (2012). Reactive Oxygen Species in Cancer Stem Cells. Antioxid. Redox Signal..

[158] Martinez-Cruzado L., Tornin J., Santos L., Rodriguez A., García-Castro J., Morís F., Rodriguez R. (2016). Aldh1 Expression and Activity Increase During Tumor Evolution in Sarcoma Cancer Stem Cell Populations. Sci. Rep..

[159] Ikeda J.-I., Tanaka H., Ishikawa K., Sakakita H., Ikehara Y., Hori M. (2018). Plasma-activated medium (PAM) kills human cancer-initiating cells. Pathol. Int..

[160] Lee Y.J., Kim S.W., Jung M.H., Kim Y.S., Kim K.S., Suh D.S., Kim K.H., Choi E.H., Kim J., Kwon B.S. (2022). Plasma-activated medium inhibits cancer stem cell-like properties and exhibits a synergistic effect in combination with cisplatin in ovarian cancer. Free. Radic. Biol. Med..

[161] Han I., Choi E.H. (2017). The role of non-thermal atmospheric pressure biocompatible plasma in the differentiation of osteoblastic precursor cells, MC3T3-E1. Oncotarget.

